# Development and validation of a food frequency questionnaire in adult Saudi subjects in Jeddah city

**DOI:** 10.1186/s12889-023-17511-9

**Published:** 2024-01-02

**Authors:** Sarah M. Ajabnoor, Hanan Jambi, Suhad Bahijri

**Affiliations:** 1 Clinical Nutrition Department, Faculty of Applied Medical Sciences, King Abdulaziz University, Jeddah, Saudi Arabia; 2 Food, Nutrition and Lifestyle Unit, King Fahd Medical Research Centre, King Abdulaziz University, Jeddah, Saudi Arabia; 3 Saudi Diabetes Research Group, Deanship of Scientific Research, King Abdulaziz University, Jeddah, Saudi Arabia; 4 Department of Food and Nutrition, Faculty of Human Sciences and Design, King Abdulaziz University, Jeddah, Saudi Arabia; 5 Department of Clinical Biochemistry, Faculty of Medicine, King Abdulaziz University, Jeddah, Saudi Arabia

**Keywords:** Food frequency questionnaire, FFQ, Dietary intake, Saudi, Adults

## Abstract

**Background and aims:**

In Saudi Arabia, very limited studies have been conducted to evaluate the validity of culturally appropriate food frequency questionnaire (FFQ). The aim of this study was to validate a newly designed FFQ against two reference methods in Saudi adults.

**Methods:**

A new FFQ adapted from the Block FFQ was completed via interview and validated against three-day food records (3DFRs; *n* = 126) and 24-hour urinary urea nitrogen (UUN)-based protein intake estimates (*n* = 118) in adult Saudis living in Jeddah. FFQ-estimated nutrient intake was compared to the 3DFR and UUN methods using Pearson’s correlations (*r*), Bland–Altman plots, and weighted kappa (*κ*_w_) statistics.

**Results:**

This study included 126 participants (80 females and 46 males). The FFQ generally overreported nutrient intakes compared to the reference methods. The FFQ was strongly correlated with 3DFRs for energy, protein, carbohydrate, and total fat (*r* > 0.7); moderately correlated with cholesterol (*r* = 0.55) and iron (*r* = 0.44); and weakly correlated with the other micronutrients (*r* = 0.1–0.3). A moderate positive correlation for protein intake was found (r = 0.62) between the FFQ and 24-hour UUN method. The Bland–Altman analysis indicated the FFQ had an acceptable level of agreement with no significant proportional bias (*P* > 0.05) with the 3DFRs for energy, protein, total fat, and iron and with protein intake. Similarly, an acceptable level of agreement was found between the FFQ and the 24-hour UUN method for estimating protein intake. Cross-classification analysis showed that ≥ 50% of participants were ranked within the same quartile for energy, protein, and total fat. The FFQ showed good agreement with the 3DFRs for energy and protein (*κ*_w_ ≥ 0.61) and acceptable agreement with protein intake. An acceptable agreement was reported between the FFQ and 24-hour UUN method (*κ*_w_ = 0.56). Separate analyses of females and males showed stronger correlations and agreements between the FFQ and the two reference methods only in females.

**Conclusion:**

The developed FFQ is an effective and valid tool for assessing dietary intake in Saudi adults. However, it still requires future optimization to improve its validity.

**Supplementary Information:**

The online version contains supplementary material available at 10.1186/s12889-023-17511-9.

## Introduction

Studying dietary intakes and their relationship to health and chronic diseases in communities is important in formulating the etiological theory of disease [1]. Therefore, such studies greatly need accurate methods to assess middle or long-term dietary intake. Laboratory- or unit-based dietary methods measuring exact intakes and excretion are the most accurate, followed by home-based studies (or weighed food records) requiring daily follow-up and intake measurements [2]. However, both methods are time-consuming, expensive, require considerable resources, and require participants’ full-time commitment [3–5]. Therefore, they are unsuitable for large-scale surveys. Consequently, food frequency questionnaires (FFQs) have become popular research tools in nutrition epidemiology due to their easy implementation and data analysis [2, 6, 7]. However, their results may be misleading without proper precautions during their design. Potential factors affecting their validity include local foods, dietary habits, age, socioeconomic status, seasonal variations, and sex [7, 8]. Any newly designed FFQ must be validated for a given population before being used as a dependable tool.

Self-completed food records are commonly used and acknowledged as an appropriate reference method for validating FFQs [2, 3]. Studies have reported strong and significant correlations between calculated intakes and an acceptable level of similarity when classifying subjects into different intake quantiles using these methods [9–12]. However, both FFQs and food records share the same type of error by underreporting energy intakes (EIs) when compared to calculated energy requirements [13–15]. Various studies reported that underreporting of EI increased with body mass index (BMI) [16]. Therefore, in an attempt to correct for this limitation, the EI-to-basal metabolic rate (BMR) ratio is calculated to measure the degree of energy underreporting, with various equations used to calculate the BMR and various cutoffs used to define under-reporting [17–20]. However, the cutoff for excluding implausible reported intakes remains debated [21], making another method with measurement errors uncorrelated with those of the FFQ error preferable. This issue has led to dietary intake biomarkers being used as an additional method for ascertaining intake in validation studies [22–25]. Urinary nitrogen excretion reflects protein intake and has been used in various validation studies [22, 24, 26, 27].

To our knowledge no adequately validated FFQ exists for Saudi adults despite the great need for one. Therefore, we aimed to design such an FFQ by adapting the Block FFQ to include local foods and validate it against three-day FRs (3DFRs) and 24-hour urinary nitrogen excretion to reflect protein intake.

## Methods

### Designing the questionnaire

**Fig. 1:**
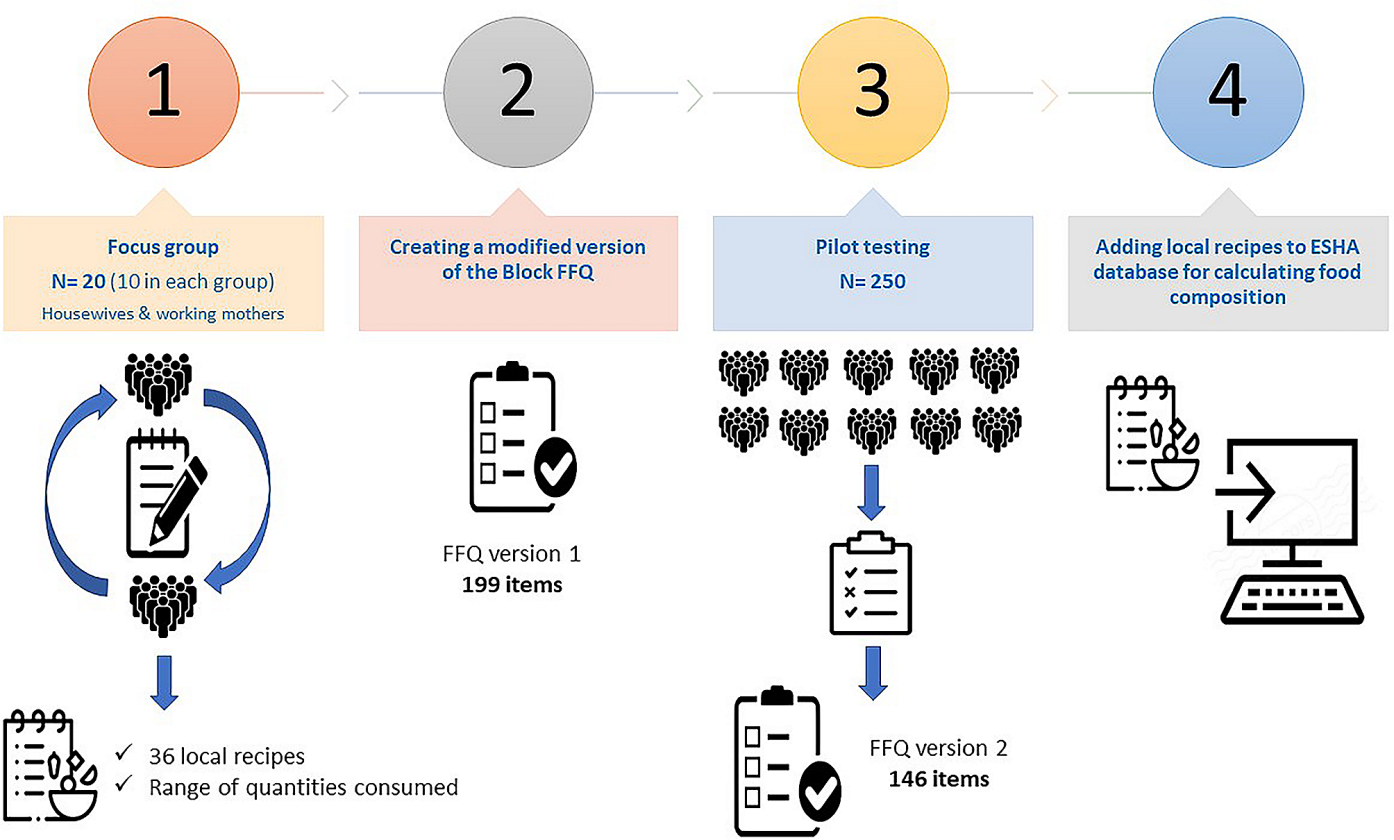
FFQ designing steps

The Block FFQ [28] was adopted and modified in several steps (Fig. 1):

#### Step one

Two focus groups sessions were conducted at King Fahad Medical Research Centre (KFMRC). The selection criteria were middle-aged working females with children living with them in the same house. The discussions were facilitated by two members of the research group. Each group discussion was recorded (with participants’ consent) and then transcribed to analyze for emerging themes. In the first group, 10 participants were recruited by direct invitation, they were mothers working in various sectors at King Abdulaziz University (KAU). The second group (n = 10) was recruited from the local community by direct invitation, they were middle-aged housewives with children living with them in the same house. At the beginning of each group discussion, the objectives of the research were explained by the facilitator then asked the participant open-ended questions probing deeper into their responses to encourage them to share their history and experiences about usual family meals and snack intake and the most common dishes at each meal. The emerging themes were found to be aligned with categories of Block FFQ, and 36 local recipes were developed and added to Food Processor Nutrition Analysis software V.11.2 (ESHA Research, USA).

#### Step two

Using the information from Step One, the new Saudi FFQ was designed by including 11 categories adopted from the Block FFQ’s general design to create a provisional version (version 1) that also included local and seasonal dishes, such as those eaten during the holy month of Ramadan, which is the ninth month of the Muslim year, during which strict fasting is observed from down to sunset. A section was added to the intake frequency to include a span of the previous year. Open-ended sections were provided at the end of each category to capture additional foods suggested by research participants during pilot testing. This version of the FFQ included 199 items, with portion size specified as indicated in the Block FFQ.

#### Step three

Version 1 of the FFQ was administered to 50 male and 50 female university medical students and 50 female and 50 male relatives and staff members working at the King Fahd Medical Research Center in Jeddah. In addition, 50 mothers were asked to complete it for their children. Items found to be unpopular (chosen by < 1% of the tested subjects) were removed, and new items suggested by ≥ 5% of responders were added to produce a second version (version 2) of the questionnaire with fewer items (146 items).

#### Step four

Additional recipes used to prepare local dishes to those collected in Step One were collected by university students from their mothers or relatives to obtain multiple versions for each recipe. In addition, all books and webpages of local recipes were consulted to compile the final version of the included recipes and added to the database in the Food Processor Nutrition Analysis software to be used to calculate nutrient composition and caloric intake. Finally, the food compositions for international fast foods were obtained from the Food Processor Nutrition Analysis software and verified from the different company websites. Items from the list chosen only by ≤ 5% of participants were excluded. The final version contained 146 items.

### Description of the designed FFQ (version 2)

Version 2 of the FFQ contained 146 items grouped into 12 categories as follows: 12 items for breakfast categories; 16 items in milk and dairy; 12 items in fruits; 13 items in vegetables; six items in soups; 10 items in pastries and baked products; eight items in fast food; 11 items in meat and fish; 12 items in bread, rice, and pasta; nine items in spreads and sauces; 14 items in snacks; and 23 items in drinks and beverages.

Visual cards of actual food serving sizes and food models from the National Dairy Council were used to help the participants identify the portion size of their consumed food. The medium portion size from the Block model was used as the reference for our FFQ. For each item, the participants showed their consumed portion size over the past year by checking one of nine frequency categories ranging from never to once or ≥ 2 times per day, 1–6 times per week, 1–3 times per month, and 1–6 times per year. Each selected frequency for each item was then converted to a daily intake by multiplying the medium serving size shown in the FFQ by the following values for each option: Never = 0; 1/day = 1; 2+/day = 2; 1/week = 0.14; 2–4/week = 0.43; 5–6/week = 0.79; 1/month = 0.033; 2–3/month = 0.08; 1–6/year = 0.01 [29].

### Validation study design

#### Study population

Healthy individuals aged 20–54 years were recruited via convenient sampling method from the university student population and their families and friends at King Abdulaziz University in Jeddah. Those taking any nutritional supplements or on special diets were excluded. Data was collected between May 1, 2013, and June 30, 2013. This study was part of a large project studying diabetes prevalence and associated factors. This study was approved by the Biomedical and Research Committee of the Faculty of Medicine at King Abdulaziz University (reference no.: 338 − 10). All participants provided their informed consent prior participating in the study.

#### Reference methods

The final version of the FFQ (version 2) was administered by interview to 80 females and 46 males. In addition, food records were collected from all participants. Each participant was given a small booklet containing instructions and pages to record foods eaten during seven periods (before breakfast, breakfast, mid-morning, lunch, tea, evening meal, and later in the evening) for three days. They were asked to enter food records for two random weekdays and one random weekend day using open-ended 3DFRs in the provided diary.

Urine containers were given to participants willing to collect 24-hour urine to measure their urinary urea and creatinine. Urinary urea was estimated spectrophotometrically by the diacetyl-monoxime method [30, 31] and converted to urinary urea nitrogen (UUN) excretion (g/d). Protein intake was calculated using the formula 6.25 × (urinary nitrogen + 2) [22] based on the assumption that urea nitrogen excretion is a constant proportion (85%) of total urinary nitrogen [26]. Creatinine was estimated using the Jaffe reaction [32]. Calculated 24-hour creatinine values were used to monitor the adequacy of sample collection [33].

#### Calculation of calories and nutrient intakes

Each selected frequency for each item consumed in the FFQ was converted into daily intake by multiplying the standard portion size of each food by the following codes for each frequency option: never = 0, once/day = 1, ≥ 2 times/day = 2, once/week = 0.14, 2–4 times/week = 0.43, 5–6 times/week = 0.79, once/month = 0.033, 2–3 times/month = 0.08, and 1–6 times/year = 0.01. Seasonal foods were weighted according to the proportion of the year that each food was available.

Data from FFQ and 3DFRs were entered into a database in the Food Processor Nutrition Analysis software, and daily intakes were calculated for energy (kcal), protein (g and % kcal), carbohydrate (g and % kcal), fat (g and % kcal), saturated fat (g), monounsaturated fat (g), polyunsaturated fat (g), cholesterol (mg), dietary fiber (g), vitamin A (RE), vitamin C (mg), calcium (mg), iron (mg), total sugar (g). The mean daily intake of the three dietary records was used to represent the 3DFRs calculated by the Food Processor Nutrition Analysis software for each participant.

#### Calculation of the EI:BMR ratio

In order to assess the degree of energy misreporting, the EI:BMR ratio was calculated from the reported EI in the FFQ and 3DFRs and the predicted BMR. First, the BMR was calculated for all participants in the validation study based on the Hayter and Henry equation [34], the most predictive equation for estimating the BMR in healthy young women [35]. Then, the EI estimated from FFQ and 3DFR was divided by the estimated BMR, creating a measure of relative EI that accounts for age, sex, and body weight. Low energy reporters were defined as participants with an EI:BMR of < 1.1 [17].

### Statistical analysis

Continuous variables (e.g., sample characteristics and the daily nutrient intakes estimated with the three dietary assessment tools) were assessed for normality using the Shapiro–Wilk test and are presented as means ± standard deviations. Categorical variables are presented as frequencies and percentages. Characteristics were compared between males and females using the Mann–Whitney U and Chi-square tests.

Food intake estimates from the FFQ and 3DFRs were compared using Pearson’s correlation coefficients (*r*) and Bland–Altman analysis [36]. In order to compare the FFQ with the mean of 3DFRs, participants were classified into quartile categories for energy and macronutrient intake based on the data distributions from the test and reference methods. The proportions of participants classified into the same, adjacent, or non-adjacent quartiles were derived from crude and energy-adjusted cross-classifications.

In addition, UUN-based protein intake was correlated with that from both dietary assessment methods. In order to compare the dietary method with the UUN method, participants were classified into quartiles for energy and protein intake according to the data distributions from the test and reference methods. The proportions of participants classified into the same, adjacent, or extreme quartiles were determined.

The weighted kappa (*κ*_w_) statistics were used to evaluate the level of agreement between the FFQ and the reference methods in classifying participants into different quartiles. A good outcome was defined as ≥ 50% of measurements were in the same quartile and ≤ 10% were in the non-adjacent quartile, and a poor outcome was defined as < 50% were in the same quartile and > 10% were in the opposite quartile [37]. The following *κ*_w_ ranges were used to assess the level of agreement: ≥0.61, good agreement; 0.20–0.60, acceptable agreement; <0.20, poor agreement [37]. All analyses were conducted using the Statistical Package for the Social Sciences software (version 25; SPSS Inc., USA). All results with a two-tailed *P*-value of < 0.05 were considered statistically significant.

## Results

This study included 126 participants (80 females and 46 males). All participants completed the FFQ and 3DFR. However, only 118 participants provided 24-hour urine samples for estimating 24-hour UUN. The study participants’ characteristics are provided in Table 1. Their mean age was 20.41 ± 2.66 years (20.48 ± 2.88 years for females and 20.3 ± 2.26 years for males). Their mean BMI was 22.68 ± 4.04 kg/m^2^ (22.42 ± 4.06 kg/m^2^ for females and 23.12 ± 4.0 kg/m^2^ for males). Most (66.7%) had a normal BMI (70% of females and 61% of males). Male and female participants did not differ significantly in age and BMI. The study sample contained no low energy reporters based on the EI:BMR. However, the mean EI:BMR differed significantly between males and females for the FFQ and 3DFR (*P* < 0.05).

The mean daily intakes of energy, macronutrients, and micronutrients assessed by the FFQ, 3DFR, and 24-hour UUN are shown in Supplementary Tables 1 and 2. The FFQ generally overreported the intake of most nutrients except for fat and cholesterol compared to the reference methods.

Pearson’s correlation coefficients between the mean nutrient intakes with the FFQ and 3DFR are provided in Table 2. All nutrients showed significant correlations between the two dietary assessment tools in the total sample (*P* < 0.01) except for vitamins A and C. The correlation coefficients ranged between 0.13 (vitamin C) and 0.95 (total energy). In the total sample, the correlations were strongly positive (*r* > 0.7) for energy, protein, carbohydrate, and fat and moderately positive for cholesterol (*r* = 0.55) and iron (r = 0.44). However, they were only weakly positive for the other micronutrients (*r* = 0.1–0.3). When females and males were analyzed separately, the correlation coefficients increased for the following micronutrients in females: saturated fatty acids (SFAs; *r* = 0.41, *P* < 0.01), monounsaturated fatty acids (MUFAs; *r* = 0.54, *P* < 0.01), polyunsaturated fatty acids (PUFAs; *r* = 0.42, *P* < 0.01), and calcium (*r* = 0.49, *P* < 0.01). In contrast, the correlation coefficients decreased for the following nutrients in males: carbohydrates (*r* = 0.63, *P* < 0.01), fat (*r* = 0.60, *P* < 0.01), cholesterol (*r* = 0.15, *P* > 0.05), and iron (*r* = 0.02, *P* < 0.05). Protein intake estimates with the FFQ and 24-hour UUN method were moderately positively correlated (*r* = 0.62, *P* < 0.01) in the total sample (*n* = 118). The correlation was similarly strong in females (*r* = 0.63, *P* < 0.01) but weaker in males (*r* = 0.26, *P* > 0.05).

**Fig. 2:**
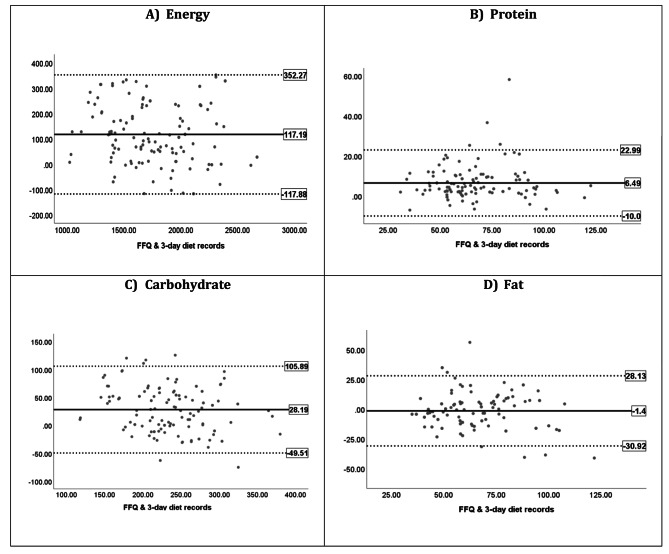
Blond Altman plots for energy and macronutrients intake with mean difference and limits of agreements. (**A**) Energy. (**B**) Protein. (**C**) Carbohydrates. (**D**) Fat. Horizontal lines represent the mean difference (solid black) and 95% limits of agreement (dotted lines)

**Fig. 3:**
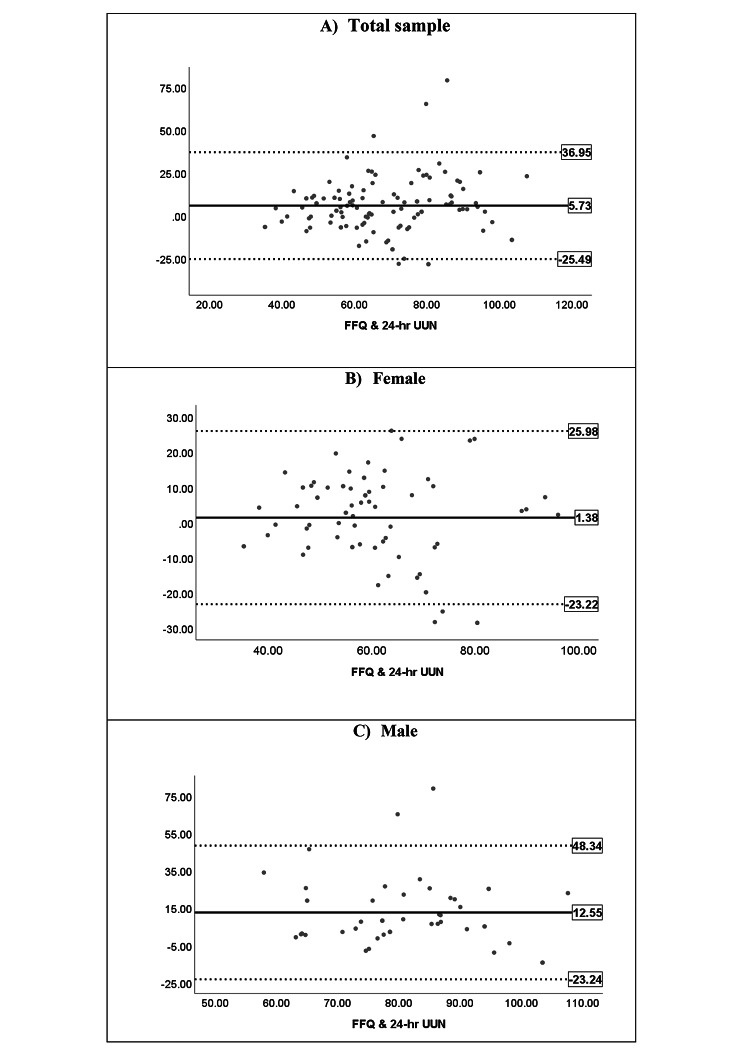
Blond Altman plots for protein intake with mean difference and limits of agreements. (**A**) Total sample (**B**) Female. (**C**) Male. Horizontal lines represent the mean difference (solid black) and 95% limits of agreement (dotted lines)

Tables 2 and 3 show the agreement between the FFQ and the 3DFR and 24-hour UUN methods, respectively. Both tables include the mean difference between methods with a 95% limit of agreement (LOA) and regression coefficients for the difference on the average. In the total sample, the FFQ overestimated most nutrients, as indicated by the positive mean difference values, but underestimated total fat, SFAs, PUFAs, and cholesterol, as indicated by the negative mean difference values. The 95% LOA was considered wide for most nutrients, possibly indicating discrepancies between the two methods for some participants. However, the 95% LOA was relatively narrower in females for some nutrients (e.g., protein, SFAs, and cholesterol). In order to visually evaluate the level of agreement, the variability of differences between each pair of methods is shown in Bland–Altman plots (Figs. 2 and 3 and Supplementary Fig. 1). Figure 2 shows that all differing points were within the LOA for energy, with a mean difference of 117.19 kcal, while only a few participants fell outside the LOA for the three macronutrients. Nevertheless, the variability of differences is relatively consistent with no observed proportional bias.

The linear regression analysis applying the regression line equation (difference between two methods = a + b [average of two methods]) found no statistically significant *t*-scores (*P* > 0.05) for energy, protein, and fat, confirming the absence of a trend (i.e., a change in size or direction of the variability of differences between the low and high scores across the plot). Therefore, an acceptable level of agreement between the two methods was shown for these nutrients. For micronutrients, most had a poor level of agreement between the two methods, as indicated by their Bland–Altman plots (Supplementary Fig. 1). Based on the linear regression findings, there was a statistically significant proportional bias for the following micronutrients: SFAs (*β* = −0.843, *P* < 0.001), PUFAs (*β* = −0.192, *P* < 0.05), cholesterol (*β* = −0.336, *P* < 0.001), vitamin A (*β* = −0.430, *P* < 0.001), calcium (*β* = −0.204, *P* < 0.05), and total sugar (*β* = −0.273, *P* < 0.01). Nevertheless, the Bland–Altman plot for iron indicated an acceptable level of agreement with no significant proportional bias (*β* = −0.085, *P* > 0.05; Supplementary Fig. 1M). When females and males were analyzed separately, the level of agreement improved for carbohydrates and remained acceptable for energy, protein, total fat, and iron in females. In contrast, the level of agreement decreased for total fat and iron but remained acceptable only for energy and protein in males.

An acceptable level of agreement was found between protein intake estimates with FFQ and the 24-hour UUN method in the total sample (*n* = 118; Table 3), with a mean difference of 5.73 g. In the Bland–Altman plot (Fig. 3), a few participants fell outside the LOA, and the mean difference was not associated with the mean of the two methods (*β* = 0.107, *P* > 0.05), confirming an absence of proportional bias with an acceptable level of agreement between the two methods. A similar trend was observed in females but with a lower mean difference (1.38 g). However, the mean difference between the two methods was relatively large in males (12.55 g) with broad LOA, indicating considerable over-reporting by the FFQ in males.

Furthermore, the cross-classification analysis of the quartiles for energy and nutrient intakes with the FFQ and 3DFR is shown in Table 4. For energy, protein, and total fat, ≥ 50% of the participants were within the same quartile. The *κ*_w_ values for energy and protein were 0.83 and 0.72, respectively, indicating a good agreement between the two methods. However, the *κ*_w_ values were lower for carbohydrates (0.48), total fat (0.59), and cholesterol (0.46), indicating an acceptable level of agreement between the two methods. However, the *κ*_w_ values ranged from 0.16 (vitamin A) to 0.28 (MUFAs, PUFAs, and calcium) for all micronutrients, with ≤ 50% of participants classified in the same quartile, indicating a poor level of agreement. When females and males were analyzed separately, the level of agreement for energy and protein remained good (*κ*_w_ ≥ 0·61) only in females. Carbohydrates and total fat showed acceptable levels of agreement in both sexes but with higher *κ*_w_ values in females. In addition, MUFAs, PUFAs, and calcium showed improved levels of agreement in females with *κ*_w_ values of 0.38, 0.44, 0.32, respectively.

An acceptable level of agreement was found between estimated protein intake quartiles with the FFQ and 24-hour UUN method (*κ*_w_ = 0.56; Table 5), with ≥ 50% of participants within the same quartile. This agreement remained acceptable when males and females were analyzed separately.

**Table 1:** Characteristics of the study population

Variable	Total (n = 126)	Female (n = 80)	Male (n = 46)	*p*-value^*^
Age (years)	20.41 ± 2.66	20.48 ± 2.88	20.3 ± 2.26	0.41
Height (cm)	163 ± 7.98	159.13 ± 4.38	169.7 ± 8.4	0.000
Weight (kg)	60.69 ± 12.85	56.84 ± 10.32	67.37 ± 14.14	0.000
BMI (kg/m^2^)	22. 68 ± 4.04	22.42 ± 4.06	23.12 ± 4.0	0.331
BMI categories				
Underweight (below 18.5) Normal (18.5–24.9) Overweight (25.0–29.9) Obese (30.0 or higher)	14 (11.1%) 84 (66.7%) 22 (17.5%) 6 (4.8%)	10 (12.5%) 56 (70%) 12 (15%) 2 (2.5%)	4 (8.7%) 28 (60.9%) 10 (21.7%) 4 (8.7%)	0.277
Predicted BMR (kJ/d)	6057 ± 885.25	5551.67 ± 485.06	6936.0.7 ± 721.02	0.000
EI:BMR^**^				
FFQ 3-day food records	1.26 ± 0.16 1.18 ± 0.17	1.24 ± 0.17 1.15 ± 0.18	1.3 ± 0.13 1.23 ± 0.12	0.024 0.018

Continuous variables presented in mean ± SD and categorical variables presented in frequency (percentage)

^*^Mann-Whitney U and Chi-square tests

^**^EI: estimated intake reported by FFQ and 3-day FR; BMR: estimated basal metabolic rate based on the Hayter & Henry (1994) equation [34]

**Table 2:** Mean difference and 95% limits on agreement (LOA) and Pearson correlation coefficients between FFQ and average of 3-day diet records (n = 126)

Energy/Nutrient	Mean difference^a^	95% LOA ^b^	β ^c^	*P* value ^d^	r ^e^
Energy (kcal)					
*Total* *Female* *Male*	117.19 117.15 117.27	-117.88–352.27 -115.53–349.83 -124.49–359.03	-0.117 -0.169 -0.127	0.192 0.133 0.399	0.95^**^ 0.93^**^ 0.88^**^
Protein (g)					
*Total* *Female* *Male*	6.49 5.48 8.26	-10.00–22.99 -7.17–18.13 -13.09–29.60	0.40 0.018 -0.200	0.655 0.873 0.182	0.90^**^ 0.88^**^ 0.75^**^
Carbohydrate (g)					
*Total* *Female* *Male*	28.19 33.37 19.18	-49.51–105.89 -40.96–107.69 -61.88–100.24	-0.231 -0.055 -0.370	0.009 0.628 0.011	0.74^**^ 0.70^**^ 0.63^**^
Total Fat (g)					
*Total* *Female* *Male*	-1.40 -3.58 2.40	-30.92–28.13 -32.69–25.53 -26.68–31.47	-0.104 -0.220 -0.311	0.245 0.050 0.035	0.71^**^ 0.65^**^ 0.60^**^
SFA (g)					
*Total* *Female* *Male*	-3.43 -1.36 -7.02	-41.05–34.19 -14.35–11.63 -66.64–52.60	-0.843 -0.418 -0.926	0.000 0.000 0.000	0.29^**^ 0.41^**^ 0.12
MUFA (g)					
*Total* *Female* *Male*	3.48 3.60 3.28	-9.44–16.41 -7.19–14.39 -12.82–19.38	-0.064 0.043 -0.299	0.478 0.708 0.044	0.39^**^ 0.54^**^ -0.38^**^
PUFA (g)					
*Total* *Female* *Male*	-0.19 0.49 -1.36	-10.59–10.22 -8.34–9.31 -13.83–11.11	-0.192 0.206 -0.639	0.032 0.067 0.000	0.29^**^ 0.42^**^ -0.02
Cholesterol (mg)					
*Total* *Female* *Male*	-27.74 -15.55 -48.94	-260.65–205.17 -171.46–140.36 -373.33–275.45	-0.336 -0.539 -0.232	0.000 0.000 0.121	0.55^**^ 0.51^**^ 0.15
Fiber (g)					
*Total* *Female* *Male*	4.16 4.04 4.37	-6.78–15.10 -6.54–14.62 -7.28–16.03	0.046 -0.083 0.245	0.611 0.462 0.101	0.32^**^ 0.33^**^ 0.31^*^
Vit. A (RE)					
*Total* *Female* *Male*	293.44 327.16 234.8	-1338.30–1925.18 -1321.89–1976.21 -1377.90–1847.51	-0.430 -0.604 -0.159	0.000 0.000 0.291	0.17 0.08 0.19
Vit. C (mg)					
*Total* *Female* *Male*	67.99 75.52 54.89	-94.49–230.46 -82.32–233.36 -114.02–223.79	0.131 0.366 -0.178	0.143 0.001 0.237	0.13 0.1 0.21
Calcium (mg)					
*Total* *Female* *Male*	128.14 130.74 123.62	-412.71–668.99 -233.76–495.24 -637.42–884.66	-0.204 -0.263 -0.180	0.022 0.018 0.232	0.23^**^ 0.49^**^ -0.01
Iron (mg)					
*Total* *Female* *Male*	1.75 1.86 1.55	-4.22–7.72 -3.52–7.24 -5.38–8.48	-0.085 -0.144 0.058	0.346 0.203 0.702	0.44^**^ 0.53^**^ 0.02
Total Sugar (g)					
*Total* *Female* *Male*	24.37 31.3 12.32	-66.10–114.85 -46.12–108.72 -94.05–118.70	-0.273 0.259 -0.642	0.002 0.020 0.000	0.36^**^ 0.28^*^ 0.39^**^

SFA: saturated fatty acids; MUFA: monounsaturated fatty acids; PUFA: polyunsaturated fatty acids

^a^ Mean of the difference between FFQ and Average of 3-day diet records

^b^ LOA Level of Agreement determined as mean difference ± 1.96 × SD of the difference

^c^ Slope of average of methods regressed on difference between methods (β = 0, α = 0.05)

^d^ Statistical significance of β

^e^ Pearson correlation coefficients

^*^P < 0.05

^**^P < 0.01

**Table 3:** Mean agreement and 95% limits on agreement (LOA) and Pearson correlation coefficients for protein intake between FFQ and average of 3-day diet records versus 24-hour UUN method (n = 118)

All (n = 118)					
Nutrient	Mean difference^a^	95% LOA ^b^	β ^c^	*P* value ^d^	r ^e^
Protein (g) (3-day FR vs. 24-hr UUN)	-0.81	-30.07–28.45	0.107	0.249	0.66^**^
Protein (g) (FFQ vs. 24-hr UUN)	5.73	-25.49–36.95	0.130	0.160	0.62^**^
**Female (n = 72)**					
**Nutrient**	**Mean difference** ^**a**^	**95% LOA** ^**b**^	**β** ^**c**^	***P*** **value**^**d**^	**r** ^**e**^
Protein (g) (3-day FR vs. 24-hr UUN)	-4.07	-27.31–19.17	-0.181	0.127	0.66^**^
Protein (g) (FFQ vs. 24-hr UUN)	1.38	-23.22–25.98	-0.147	0.218	0.63^**^
**Male (n = 46)**					
**Nutrient**	**Mean difference** ^**a**^	**95% LOA** ^**b**^	**β** ^**c**^	***P*** **value**^**d**^	**r** ^**e**^
Protein (g) (3-day FR vs. 24-hr UUN)	4.29	-30.43–39.01	0.033	0.827	0.38^**^
Protein (g) (FFQ vs. 24-hr UUN)	12.55	-23.24–48.34	-0.107	0.478	0.26

3-dayFR: 3-day food records; FFQ: food frequency questionnaire; 24-hr UUN: 24 h urinary urea nitrogen

^a^ Mean of the difference between the two dietary assessment methods

^b^ LOA Level of Agreement determined as mean difference ± 1.96 × SD of the difference

^c^ Slope of average of methods regressed on difference between methods (β = 0, α = 0.05)

^d^ Statistical significance of β

^e^ Pearson correlation coefficients

^*^P < 0.05

^**^P < 0.01

**Table 4:** Cross-classification analysis in quartiles of energy and nutrient intakes between food frequency questionnaire and 3-day food records

Energy/Nutrient	Same quartile (%)	One quartile apart (%)	Misclassified (%)	Weighted Kappa
Energy (kcal)				
*Total* *Female* *Male*	78.6% 81.3% 73.9%	21.4% 18.8% 26.1%	0% 0% 0%	0.83 0.81 0.59
Protein (g)				
*Total* *Female* *Male*	69% 66.3% 73.9%	27.8% 31.3% 21.7%	3.2% 2.5% 4.3%	0.72 0.64 0.58
Carbohydrate (g)				
*Total* *Female* *Male*	46% 45% 47.8%	44.4% 43.8% 45.6%	9.6% 11.3% 6.5%	0.48 0.49 0.37
Total Fat (g)				
*Total* *Female* *Male*	58.7% 58.8% 58.7%	31.8% 27.6% 39.1%	9.6% 13.8% 2.2%	0.59 0.50 0.42
SFA (g)				
*Total* *Female* *Male*	27% 27.5% 26.1%	49.2% 47.5% 52.2%	23.8% 25% 21.7%	0.21 0.10 -0.01
MUFA (g)				
*Total* *Female* *Male*	39.7% 50% 21.7%	36.5% 35% 39.1%	23.8% 15% 39%	0.28 0.38 -0.34
PUFA (g)				
*Total* *Female* *Male*	39.7% 47.5% 26.1%	39.7% 45% 30.4	20.7% 7.5% 43.5%	0.28 0.44 -0.19
Cholesterol (mg)				
*Total* *Female* *Male*	46% 45% 47.8%	42.8% 40% 47.8%	11.1% 15% 4.3%	0.46 0.28 0.19
Fiber (g)				
*Total* *Female* *Male*	33.3% 30% 39.1%	42.9% 50% 30.4%	23.8% 20% 30.3%	0.23 0.21 0.22
Vit. A (RE)				
*Total* *Female* *Male*	31.7% 30% 34.8%	39.7% 40% 39.1%	28.6% 30% 26%	0.16 0.09 0.16
Vit. C (mg)				
*Total* *Female* *Male*	30.2% 30% 30.4%	46% 45% 47.8%	23.8% 25% 21.6%	0.18 0.15 0.23
Calcium (mg)				
*Total* *Female* *Male*	42.9% 42.5% 43.5%	33.4% 35% 30.4%	23.8% 22.5% 26%	0.28 0.32 0.19
Iron (mg)				
*Total* *Female* *Male*	31.7% 32.5% 30.4%	42.9% 45% 39.1%	25.3% 22.5% 30.3%	0.26 0.26 0.04
Total Sugar (g)				
*Total* *Female* *Male*	36.5% 37.5% 34.8%	39.7% 32.5% 52.1%	23.8% 30% 12.9%	0.23 0.14 0.26

SFA: saturated fatty acids; MUFA: monounsaturated fatty acids; PUFA: polyunsaturated fatty acids

Cross-classification analyses were completed to validate the agreement between FFQ and 3-day food records in terms of proportions of participants classified into the same or ± 1 quartile apart or misclassified

**Table 5:** Cross-classification analysis in quartiles of protein intakes between the average of 3-day diet records and 24-hour UUN method and the FFQ with 24-hour UUN method

All (n = 118)				
Nutrient	Same quartile (%)	One quartile apart (%)	Misclassified (%)	Weighted Kappa
Protein (g) (3-day FR vs. 24-hr UUN)	61%	30.5%	8.4%	0.59
Protein (g) (FFQ vs. 24-hr UUN)	59.3%	28%	12.7%	0.56
**Male (n = 46)**				
**Nutrient**	**Same quartile (%)**	**One quartile apart (%)**	**Misclassified (%)**	**Weighted Kappa**
Protein (g) (3-day FR vs. 24-hr UUN)	73.9%	19.5%	6.5%	0.60
Protein (g) (FFQ vs. 24-hr UUN)	71.7%	17.3%	10.8%	0.50
**Female (n = 72)**				
**Nutrient**	**Same quartile (%)**	**One quartile apart (%)**	**Misclassified (%)**	**Weighted Kappa**
Protein (g) (3-day FR vs. 24-hr UUN)	52.8%	37.5%	9.8%	0.45
Protein (g) (FFQ vs. 24-hr UUN)	51.4%	34.7%	13.9%	0.42

3-dayFR: 3-day food records; FFQ: food frequency questionnaire; 24-hr UUN: 24 h urinary urea nitrogen

## Discussion

This study used repeated 3DFR and 24-hour UUN as reference methods to validate an interview-administered FFQ in a sample of Saudi adults. The designed FFQ overreported the intake of most nutrients except for fat and cholesterol compared to the reference methods. Strong correlations existed between the FFQ and 3DFR for energy and all macronutrients, while moderate correlations existed for cholesterol and iron. According to the validity statistics, the FFQ used in this study had an acceptable level of agreement for energy, protein, total fat, and iron compared to the 3DFR. The FFQ indicates a higher protein intake for all study participants than the 24-hour UUN method, showing an acceptable level of agreement with a low mean difference in females. Overall, the FFQ used in this study showed acceptable validity for energy and some nutrients, which improved slightly when considering only females.

This study’s results are consistent with several FFQ validation studies that found overreporting compared to diet records or 24-hour recall in energy and nutrient intakes [38, 39]. Several validation studies have investigated the correlation and agreement between FFQs and reference methods (e.g., repeated food records, 24-hour recall, or objective methods for measuring total energy expenditure or protein excretion). However, it is challenging to directly compare the findings of this study with those of other studies due to differences in the reference methods used.

The good correlations between the FFQ and 3DFR for energy and macronutrients are consistent with previous studies [40, 41]. However, the weak correlations for several micronutrients, including vitamins A and C, can be explained by seasonal variations in the availability of fruits and vegetables, which might have caused significant variations in their consumption. However, the poor correlations for SFAs, PUFAs, and calcium may reflect the challenges in estimating micronutrient intake. A recent systematic review of validation studies among adults reported low correlations between FFQs and reference methods for most fat-related nutrients [39]. In addition, it is important to note that most cooking oils available in the market are a mixture of vegetable oils, making it very difficult to assess the intake of specific fatty acids.

Creating FFQs with reliable estimates of micronutrient intake seems challenging because of the limitations associated with their use. Including the type and amount of vitamin supplements used in the FFQ’s item list has been recommended since better correlations have been reported between FFQs and reference methods for micronutrient intake in validation studies that included supplements intake in their FFQ [42]. Nevertheless, this study showed a moderate correlation and an acceptable agreement between the FFQ and 3DFR in estimating iron intake. Such findings are consistent with a previous study that reported a moderate correlation between the two methods for iron intake [43].

According to the validity statistics, the FFQ used in this study had an acceptable level of agreement for energy, protein, and total fat compared to the 3DFR. These findings are consistent with previous validation studies in Middle Eastern countries [41, 43]. However, the reported level of agreement for carbohydrates based on Bland–Altman analysis was only acceptable in females. Indeed, the Saudi diet is quite complex, with various dishes constituting different food items, making it difficult to develop a FFQ that provides valid estimations for all nutrients.

Previous validation studies have examined the agreement between FFQs and different biomarkers for estimating nutrient intake. Several reported moderate to good agreement between protein intake estimates with the FFQs and the 24-hour UUN method [22, 44–48]. Similarly, this study found an acceptable level of agreement between these two methods, suggesting that the FFQ used in this study provides reliable protein intake estimates that are consistent with the 24-hour UUN method (a well-established biomarker for measuring protein intake), particularly in females [24]. However, the FFQ tended to overestimate protein intake in males compared to the 24-hour UUN method, suggesting that it may not accurately capture protein intake in the entire population. Sex differences have been identified as a factor influencing the accuracy of dietary reporting with various dietary assessment methods [49]. Therefore, further studies are needed to validate the FFQ in individuals with different backgrounds and health conditions in the Saudi population.

This study had a few limitations. First, its small sample size and use of convenience sampling for recruitment may not have provided a representative sample of Saudi adults. However, such practice cannot be avoided even by other studies. It is logical to require trustworthy individuals with some level of literacy when applying dietary assessment methods like food records and FFQs in research. Therefore, in future studies, researchers will need to make extra efforts to assist individuals with very low levels of literacy to accurately assess their dietary intake. Second, it did not assess the reproducibility of the FFQ, making the recommendation for using this FFQ in future research inconclusive. Moreover, the study has limited external validity because it was only conducted in one region of Saudi Arabia, thus, future studies should focus on validating the FFQ over different regions. Another limitation is the gap in time between conducting the study and completing the manuscript. This might theoretically affect the pattern and quality of consumed food. However, such effects on food consumption happened mainly during the late 70 and 80 s periods (during the Oil boom period) [50] but afterward, patterns of diet were relatively stabilized. Indeed, no massive changes in individual dietary intake were noted in the last 10 years in Saudi Arabia as noted in various publications [51–53].

However, this study also had many strengths. It used the 24-hour UUN as a biomarker to measure protein intake to validate the FFQ, helping to confirm its reporting accuracy. Unlike this study, previous validation studies in Saudi Arabia used reference methods not based on objective biomarkers [54, 55]. In addition, its use of an interview-administered FFQ with visual cards of the actual food serving size and models helped avoid reporting bias. Moreover, its use of 3DFRs for separate days of the week (weekdays and weekend days) instead of 24-hour recall to validate the FFQ may have helped improve the relative validity of the FFQ by providing better estimates of nutrient intake. Finally, it considered sex differences during its analysis, which have been shown to have confounding effects on the validity of FFQs in previous studies [39, 56].

## Conclusion

The developed FFQ provided acceptable estimates for energy, protein, and total fat compared to the 3DFR. Its results are comparable to those of previous FFQs used in adult populations in other countries that have been tested for validity and reliability over a wide range of nutrients. However, the FFQ generally overestimated nutritional intakes compared to the 3DFR and 24-hour UUN methods. However, it exhibited an acceptable level of agreement with the reference methods for energy, protein, total fat, and iron intake, making it suitable for assessing absolute intakes in the Saudi population. However, its validity showed slight improvements in females. Overall, the findings of this study have many potential implications for improving dietary assessment methods used in Saudi Arabia. Future studies might consider validating the FFQ used in this study in larger samples with different age groups, which could improve validation processes and practices, ensuring that dietary assessment tools are thoroughly validated before their implementation. This study’s findings suggest that the developed FFQ may be appropriate for use in Jeddah, the second largest city in Saudi Arabia with various ethnic and cultural backgrounds, making its cuisines more internationally diverse compared to other regions of the country. Therefore, future studies must validate the FFQ in different regions of Saudi Arabia that have traditional cuisines uncommonly consumed by individuals living in Jeddah. Moreover, as new FFQs are developed and validated, future studies must evaluate their reliability to ensure the data collected is reproducible at different time points.

The generated FFQ tool from this study can be implemented in future national nutrition surveys, which can be used to evaluate diet adequacy in certain population groups that are expected to have dietary-related issues. Compared to other dietary assessment tools, the generated FFQ might be considered more convenient, economically feasible, and can be easily applicable in electronic format. Overall, the findings of this study could help in designing future dietary intervention programs, which help enhance awareness, improve nutritional status, and evaluate the later success and outcomes of such programs.

### Electronic supplementary material

Below is the link to the electronic supplementary material.

Supplementary Material 1

Supplementary Material 2

## Data Availability

The datasets used and/or analysed during the current study are available from the corresponding author on reasonable request.
