# Supplementary material for: Development and validation of a food frequency questionnaire in adult Saudi subjects in Jeddah city
Source: BMC Public Health. 2024 Jan 2;24:9. doi: 10.1186/s12889-023-17511-9 (PMC10759497; doi:10.1186/s12889-023-17511-9)
Supplement: Supplementary file 2 — Supplementary Material 2 [file 12889_2023_17511_MOESM2_ESM.docx]

**Supplementary Materials**

**Table 1: Mean of daily energy and nutrient intake estimated by the FFQ and the 3-day food record (FR)**

| **Energy/nutrient** | **Total**  **(n = 126)** | | **Female**  **(n = 80)** | | **Male**  **(n = 46)** | |
| --- | --- | --- | --- | --- | --- | --- |
|  | **FFQ** | **3-day FR** | **FFQ** | **3-day FR** | **FFQ** | **3-day FR** |
| Energy (kcal) | 1834 ± 367 | 1717 ± 381 | 1652 ± 296 | 1535 ± 316 | 2150 ± 243 | 2033 ± 259 |
| Protein (g) | 71 ± 19 | 65 ± 18 | 61 ± 13 | 56 ± 13 | 88 ± 14 | 80 ± 16 |
| Carbohydrate (g) | 250 ± 50 | 221 ± 58 | 233 ± 48 | 200 ± 50 | 279 ± 39 | 260 ± 53 |
| Total Fat (g) | 64 ± 19 | 66 ± 20 | 56 ± 16 | 59 ± 19 | 79 ± 14 | 77 ± 18 |
| SFA (g) | 21 ± 6 | 24 ± 20 | 18 ± 5 | 19 ± 7 | 25 ± 6 | 32 ± 31 |
| MUFA (g) | 18 ± 6 | 15 ± 6 | 16 ± 6 | 13 ± 6 | 21 ± 4 | 18 ± 6 |
| PUFA (g) | 9 ± 4 | 8 ± 5 | 8 ± 5 | 8 ± 4 | 10 ± 3 | 11 ± 6 |
| Cholesterol (mg) | 231 ± 103 | 258 ± 138 | 181 ± 54 | 196 ± 92 | 317 ± 111 | 366 ± 140 |
| Fiber (g) | 16 ± 5 | 12 ± 5 | 16 ± 4 | 12 ± 5 | 16 ± 6 | 11 ± 4 |
| Vit. A (RE) | 1027 ± 485 | 734 ± 763 | 942 ± 388 | 615 ± 779 | 1175 ± 594 | 941 ± 695 |
| Vit. C (mg) | 133 ± 67 | 65 ± 59 | 136 ± 70 | 60 ± 48 | 129 ± 62 | 74 ± 74 |
| Calcium (mg) | 696 ± 199 | 568 ± 243 | 669 ± 160 | 539 ± 203 | 743 ± 248 | 619 ± 297 |
| Iron (mg) | 12 ± 3 | 10 ± 3 | 11 ± 3 | 9 ± 3 | 13 ± 3 | 12 ± 2 |
| Total Sugar (g) | 114 ± 35 | 89 ± 45 | 108 ± 37 | 70 ± 28 | 124 ± 29 | 112 ± 59 |

SFA: saturated fatty acids; MUFA: monounsaturated fatty acids; PUFA: polyunsaturated fatty acids; FFQ: food frequency questionnaire; 3-dayFR: 3-day food records

Data are presented as mean ± SD

**Table 2: Mean of daily protein intake estimated by the FFQ, 3-day food record (FR), and 24-hr urine urea nitrogen**

| **Energy/nutrient** | **Total**  **(n = 118)** | | | **Female**  **(n = 72)** | | | **Male**  **(n = 46)** | | |
| --- | --- | --- | --- | --- | --- | --- | --- | --- | --- |
|  | **FFQ** | **3-day FR** | **24-hr UUN** | **FFQ** | **3-day FR** | **24-hr UUN** | **FFQ** | **3-day FR** | **24-hr UUN** |
| Protein (g) | 71 ± 19 | 65 ± 19 | 66 ± 17 | 61 ± 14 | 55 ± 13 | 60 ± 15 | 88 ± 14 | 80 ± 16 | 75 ± 16 |

FFQ: food frequency questionnaire; 3-dayFR: 3-day food records; 24-hr UUN: 24 hour urinary urea nitrogen

Data are presented as mean ± SD

**Figure 1: Blond Altman plots for energy, macronutrients, and micronutrients intake with mean difference and limits of agreements.** A) Energy. B) Protein. C) Carbohydrates. D) Total fat. E) Saturated fatty acids. F) Monounsaturated fatty acids. G) Polyunsaturated fatty acids. H) Cholesterol. I) Fiber. J) Vit. A. K) Vit. C. L) Calcium. M) Iron. N) Total sugar

| 1. **Energy** |
| --- |
| **Total Sample**  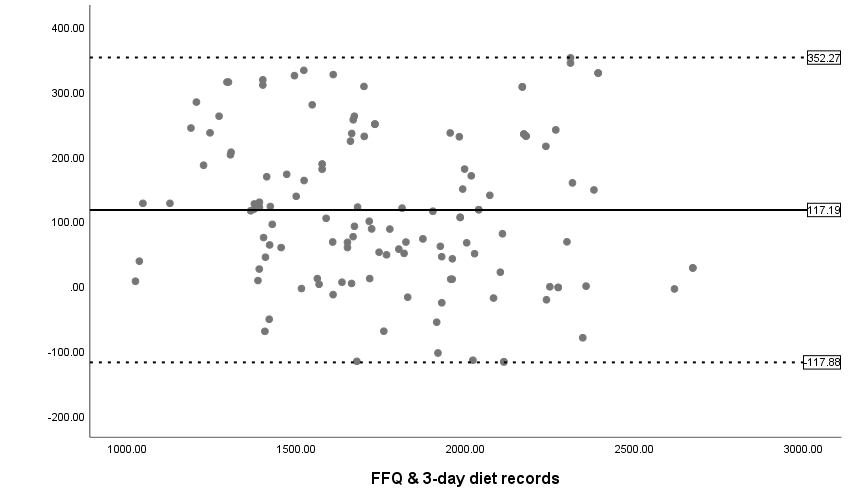 |
| **Female**  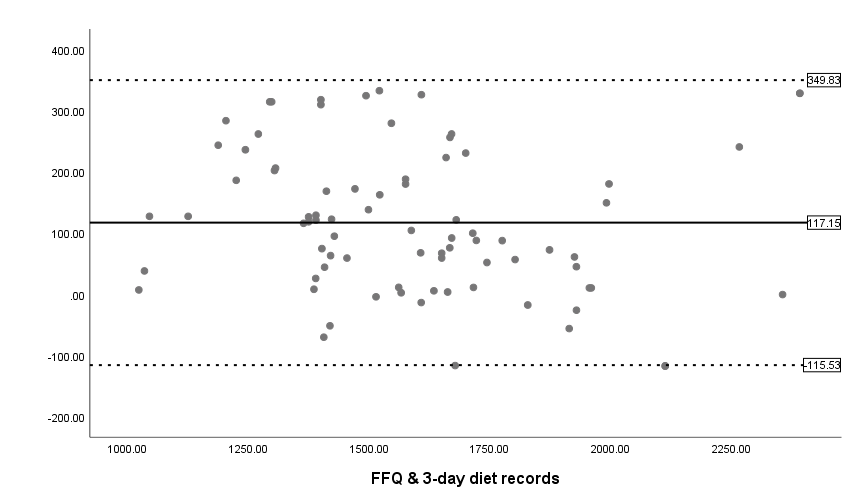 |
| **Male**  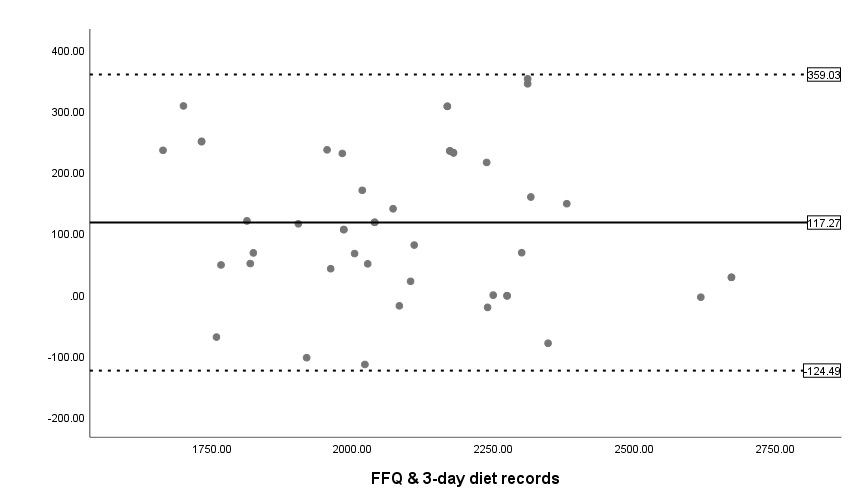 |

| 1. **Protein** |
| --- |
| **Total Sample**  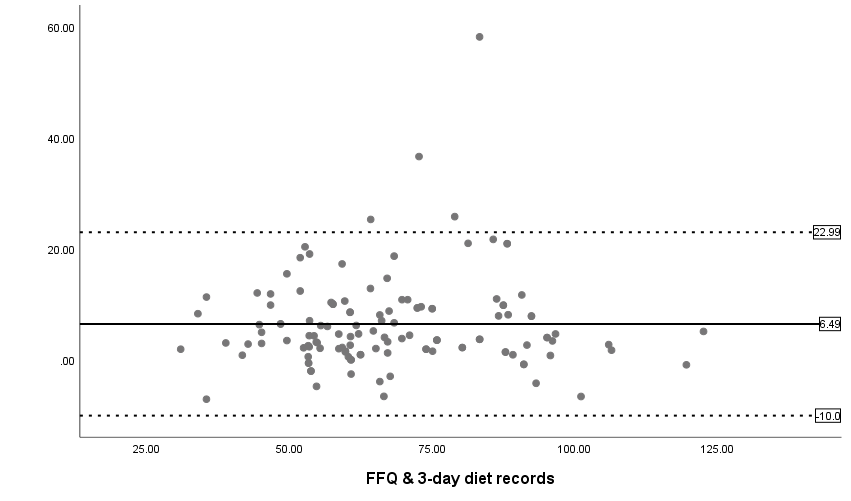 |
| **Female**  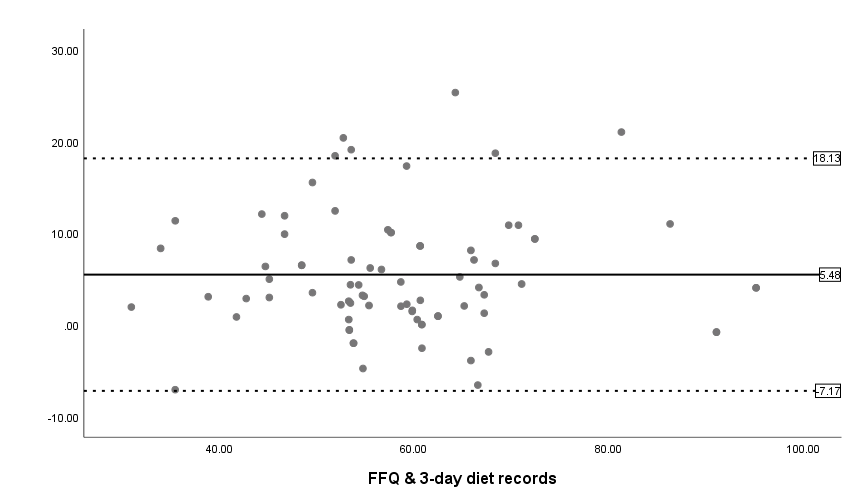 |
| **Male**  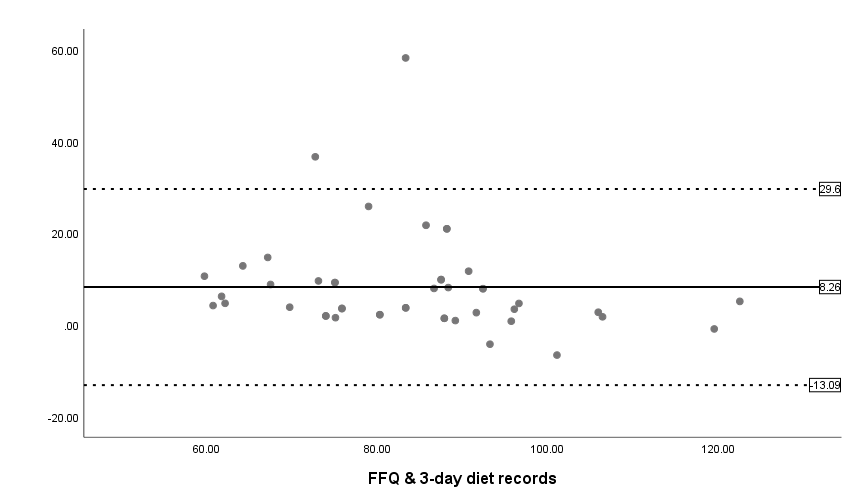 |

| 1. **Carbohydrate** |
| --- |
| **Total Sample**  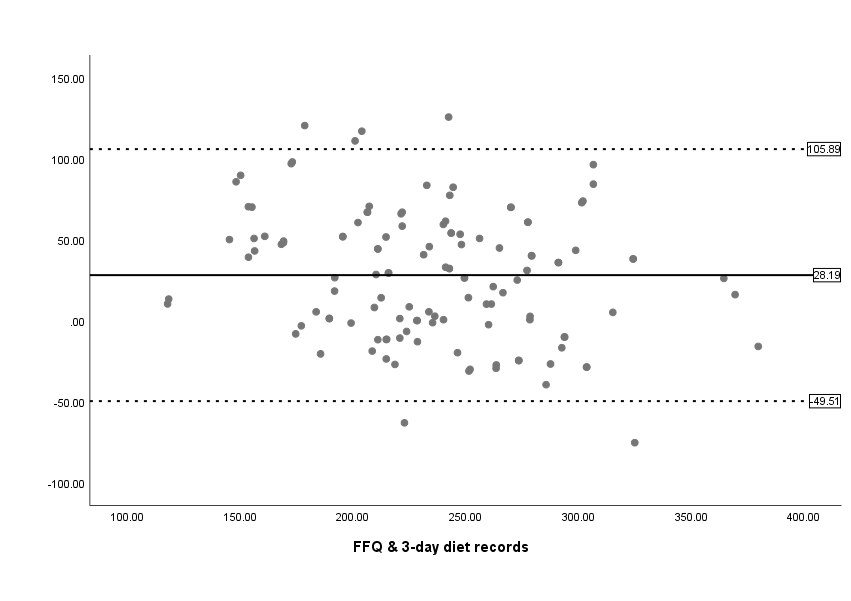 |
| **Female**  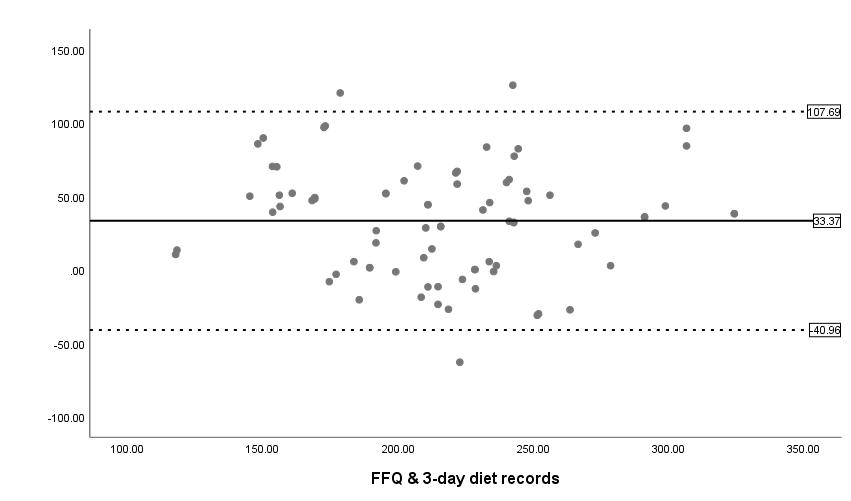 |
| **Male**  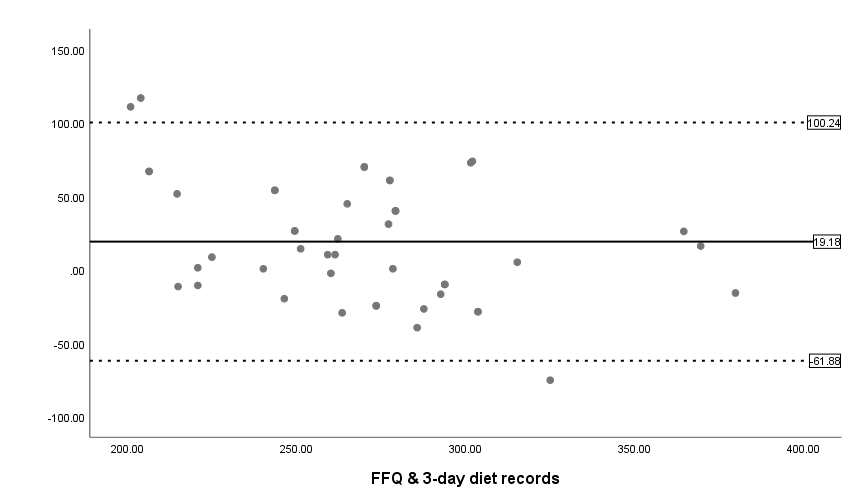 |

| 1. **Total fat** |
| --- |
| **Total Sample**  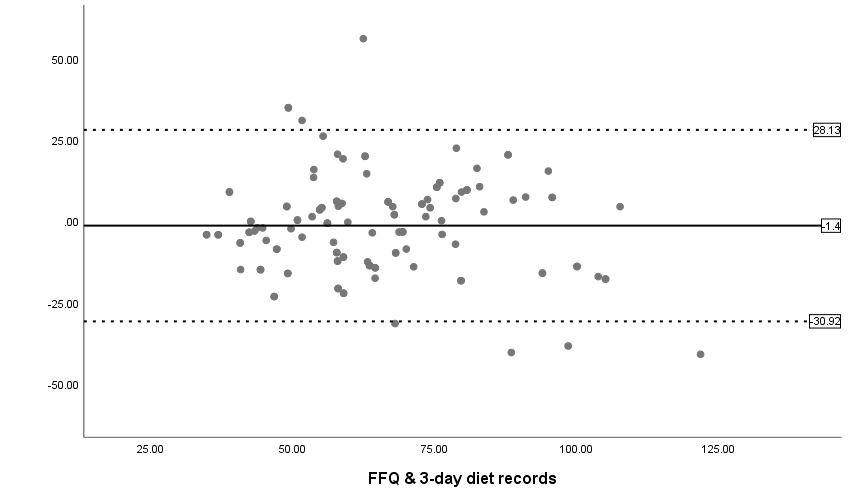 |
| **Female**  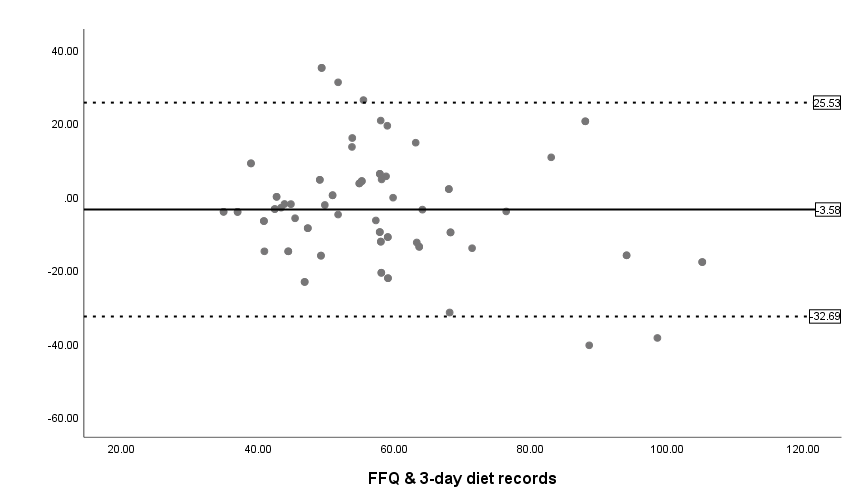 |
| **Male**  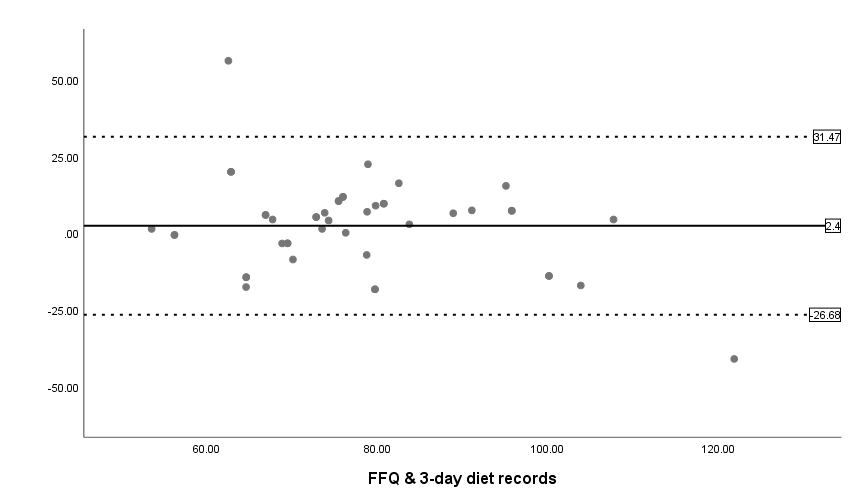 |

| 1. **Saturated fatty acids (SFA)** |
| --- |
| **Total Sample**  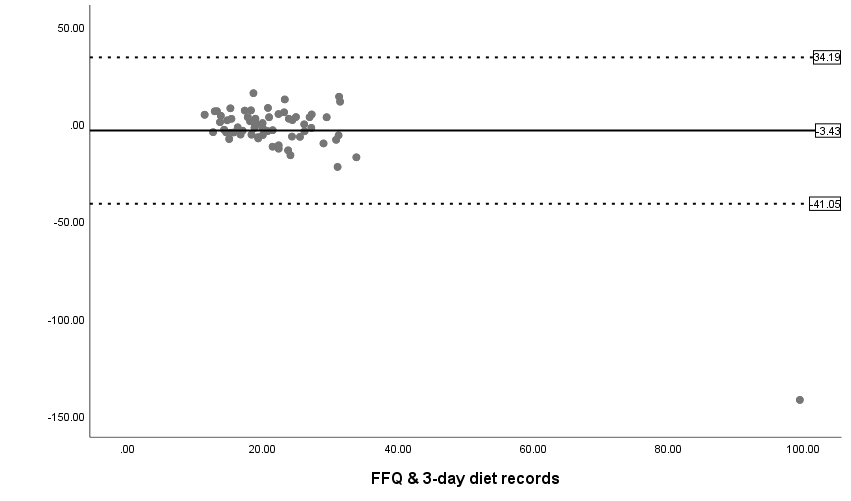 |
| **Female**  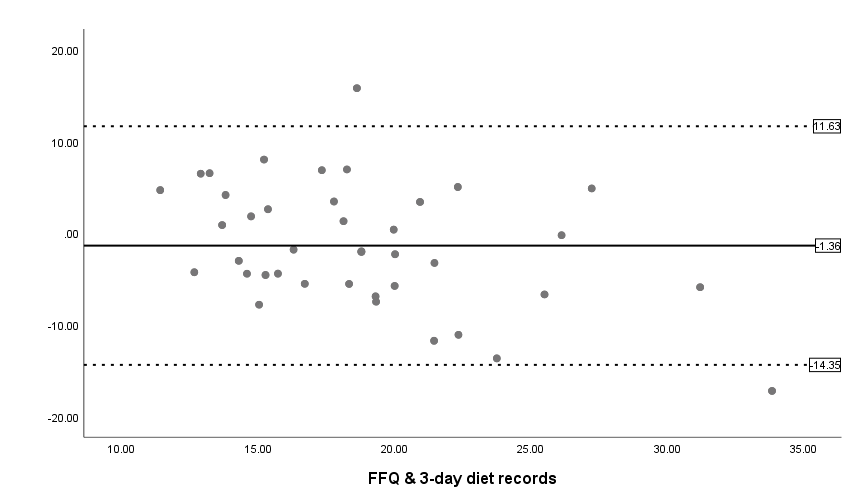 |
| **Male**  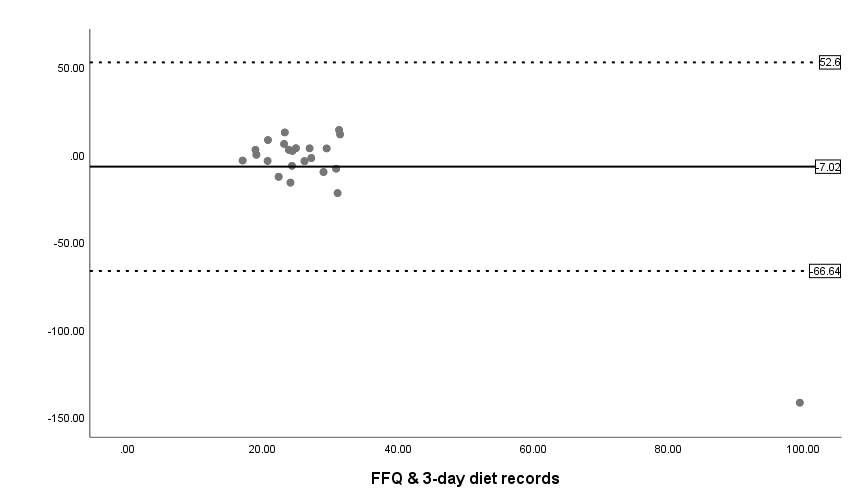 |

| 1. **Monounsaturated fatty acids** |
| --- |
| **Total Sample**  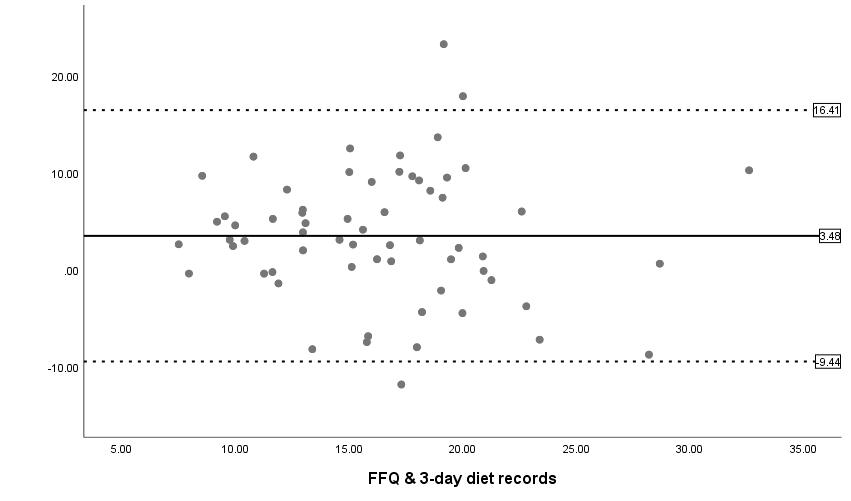 |
| **Female**  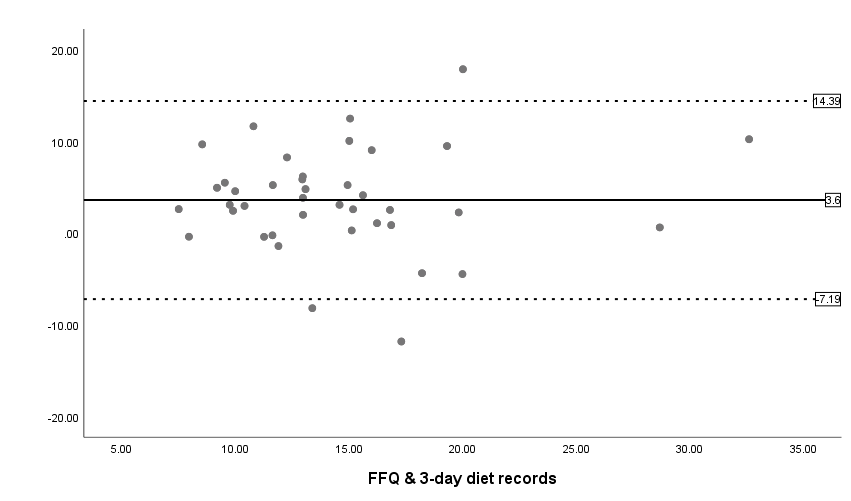 |
| **Male**  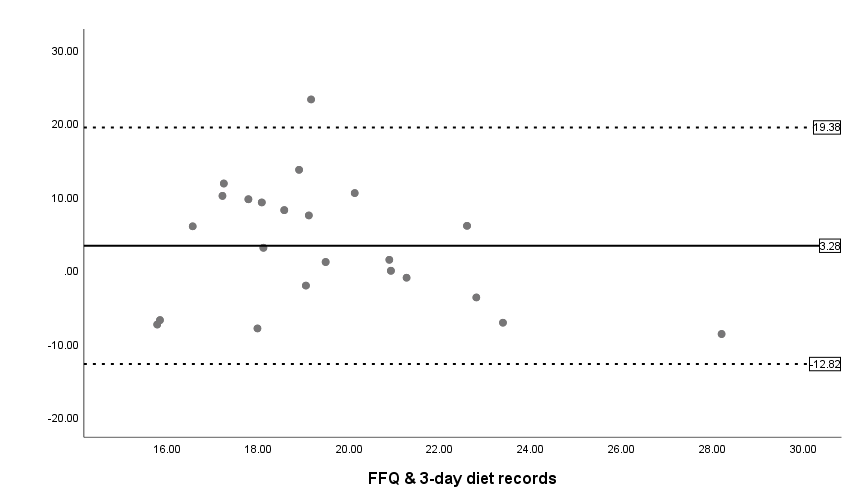 |

| 1. **Polyunsaturated fatty acids (PUFA)** |
| --- |
| **Total Sample**  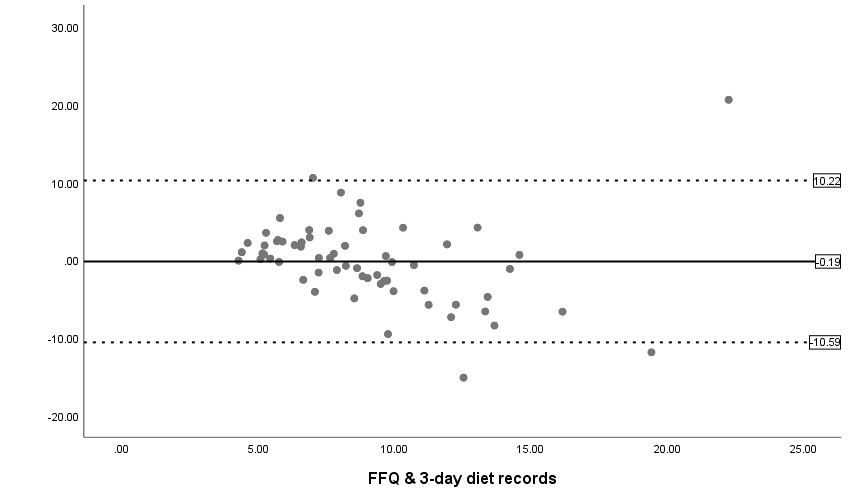 |
| **Female**  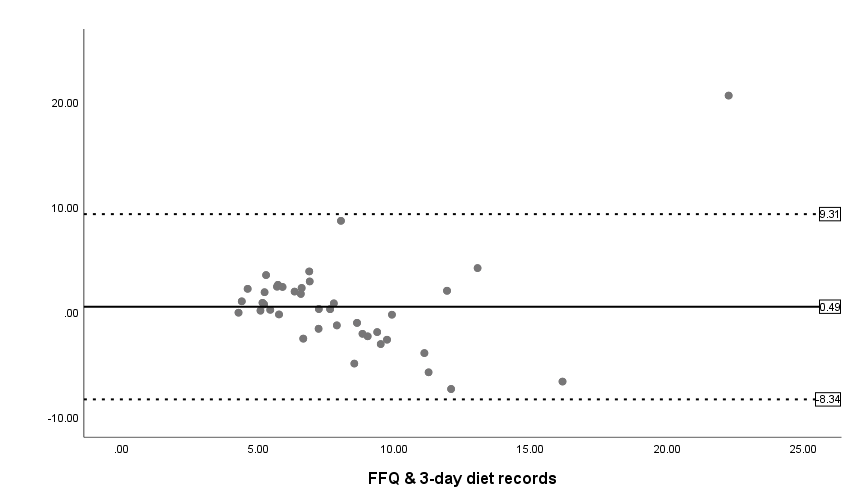 |
| **Male**  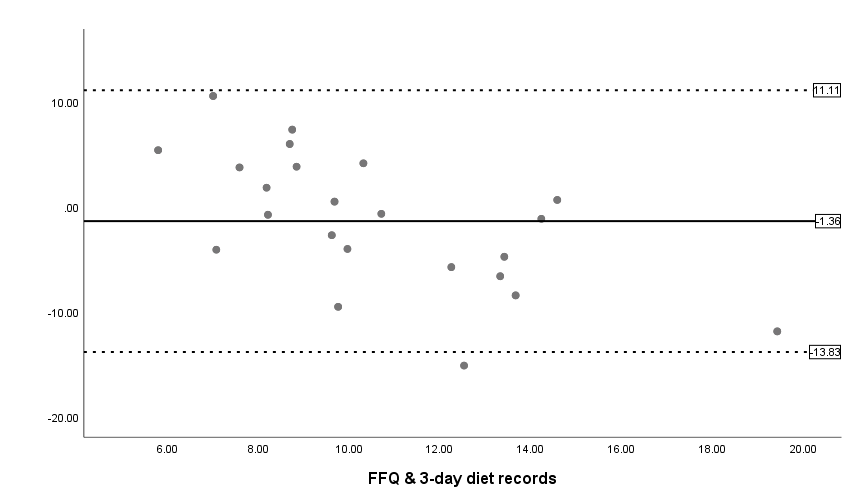 |

| 1. **Cholesterol** |
| --- |
| **Total Sample**  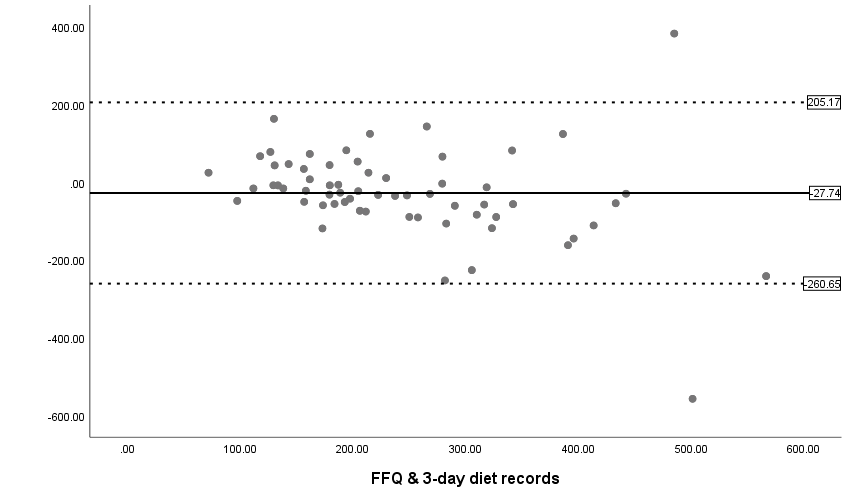 |
| **Female**  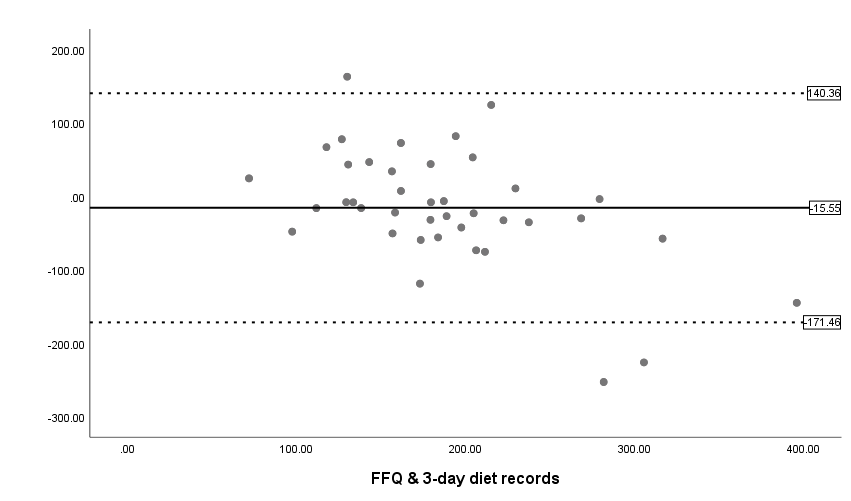 |
| **Male**  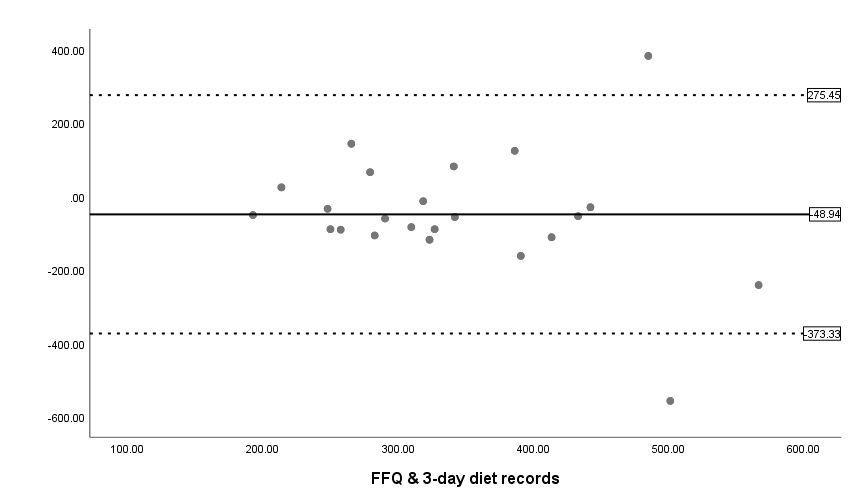 |

| 1. **Fiber** |
| --- |
| **Total Sample**  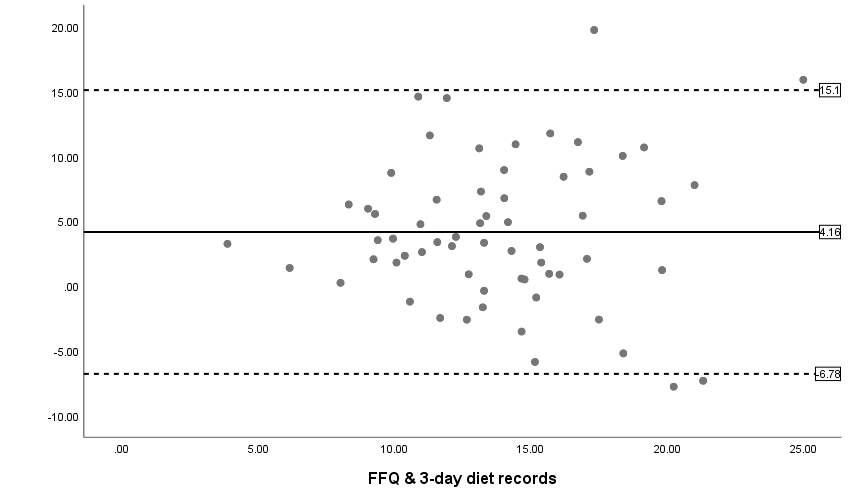 |
| **Female**  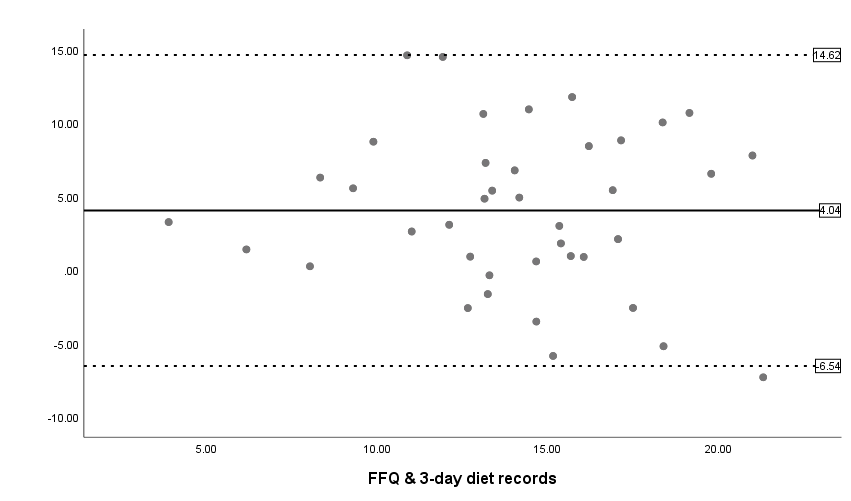 |
| **Male**  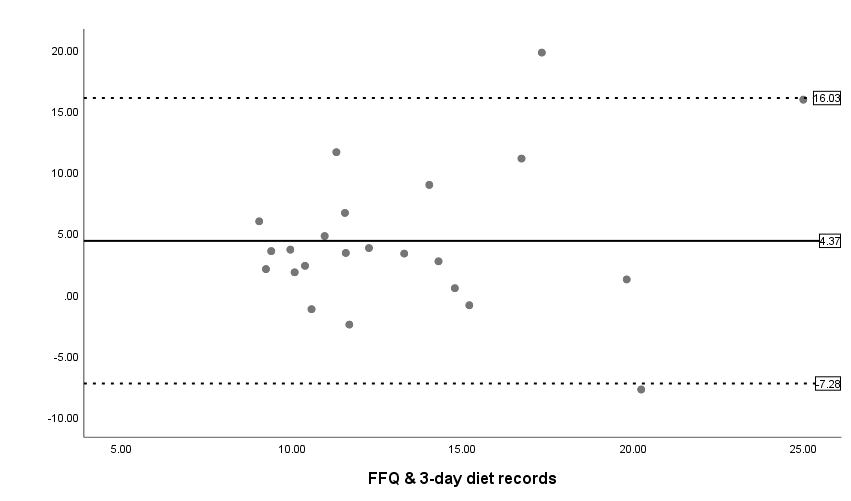 |

| 1. **Vit. A** |
| --- |
| **Total Sample**  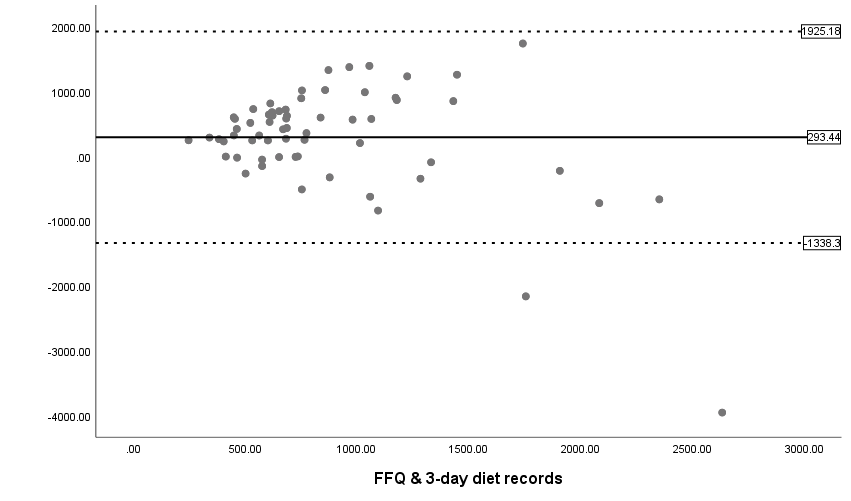 |
| **Female**  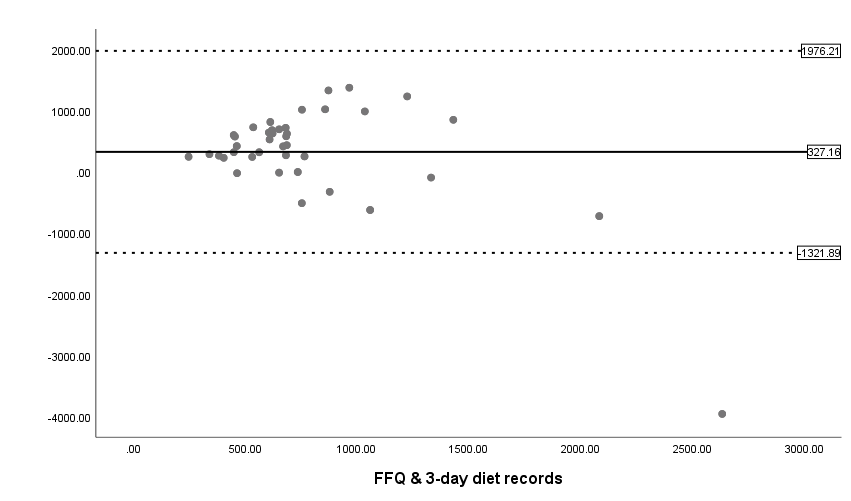 |
| **Male**  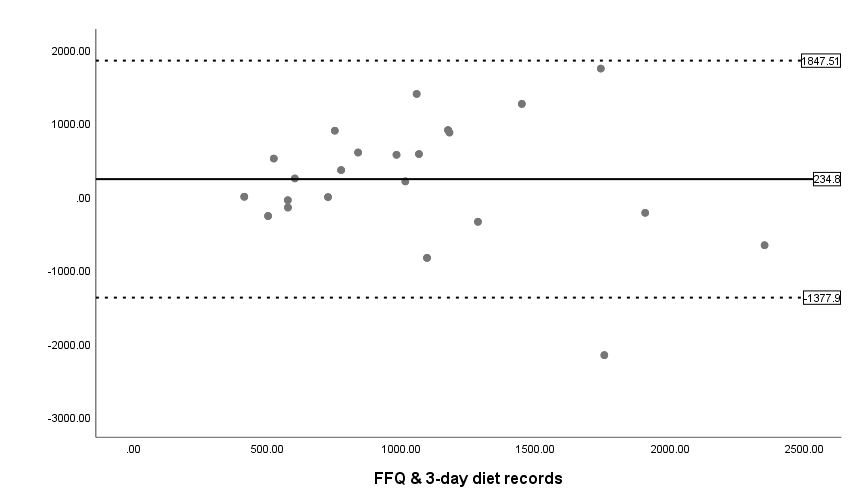 |

| 1. **Vit. C** |
| --- |
| **Total Sample**  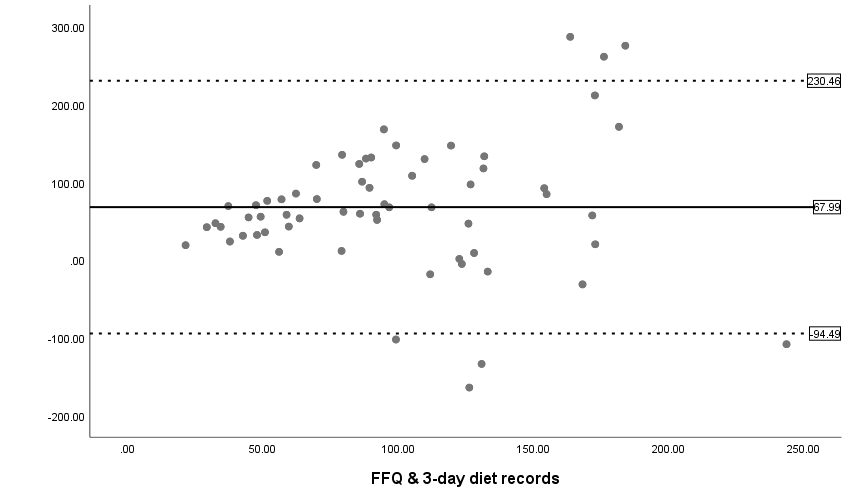 |
| **Female**  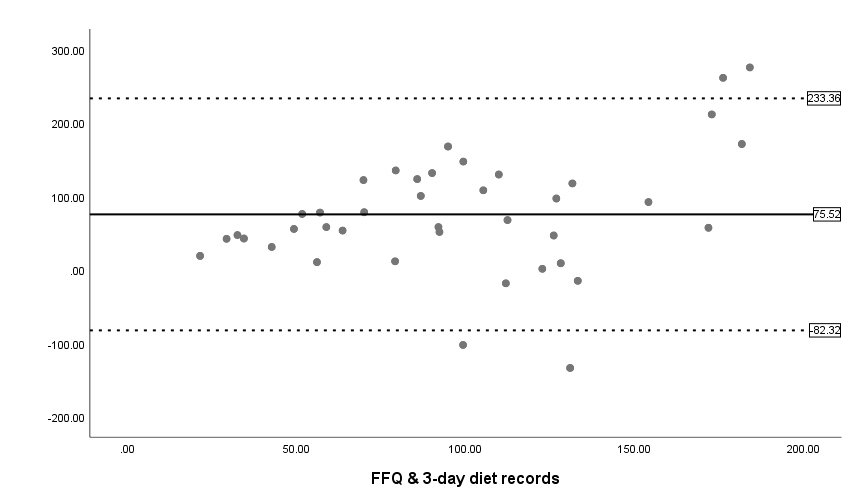 |
| **Male**  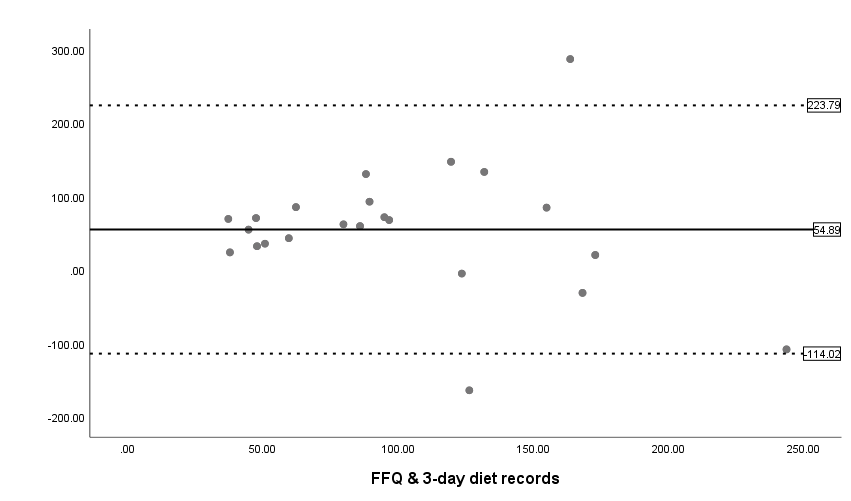 |

| 1. **Calcium** |
| --- |
| **Total Sample**  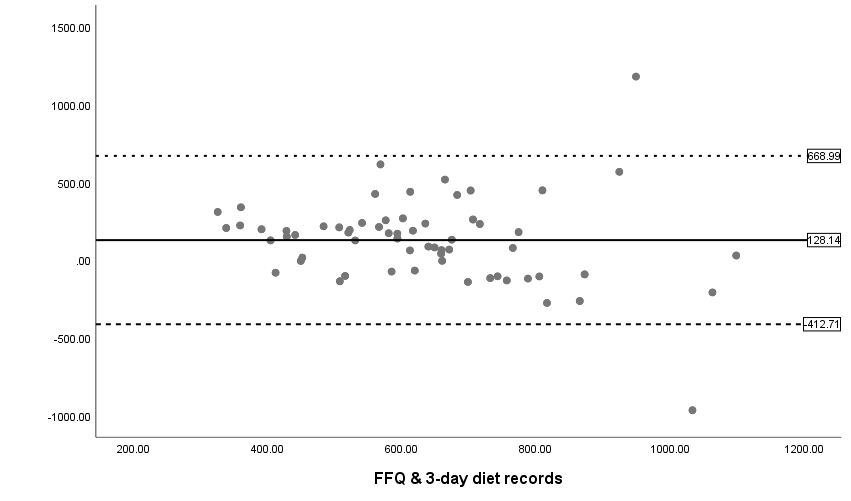 |
| **Female**  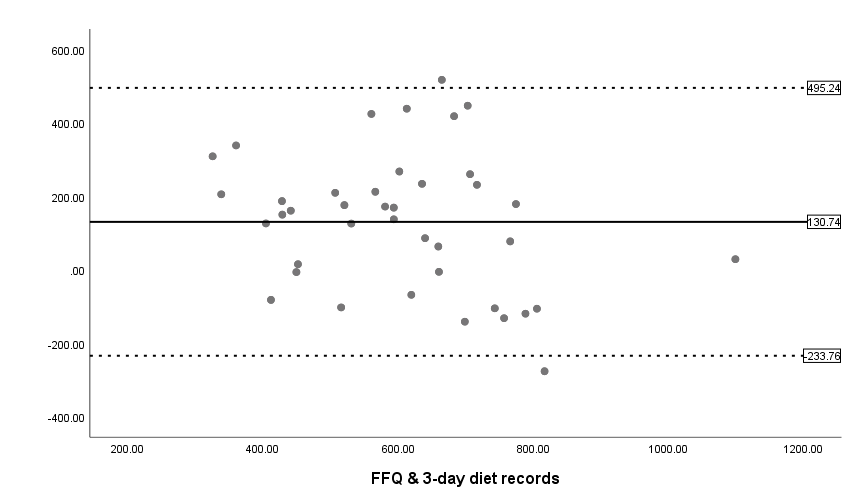 |
| **Male**  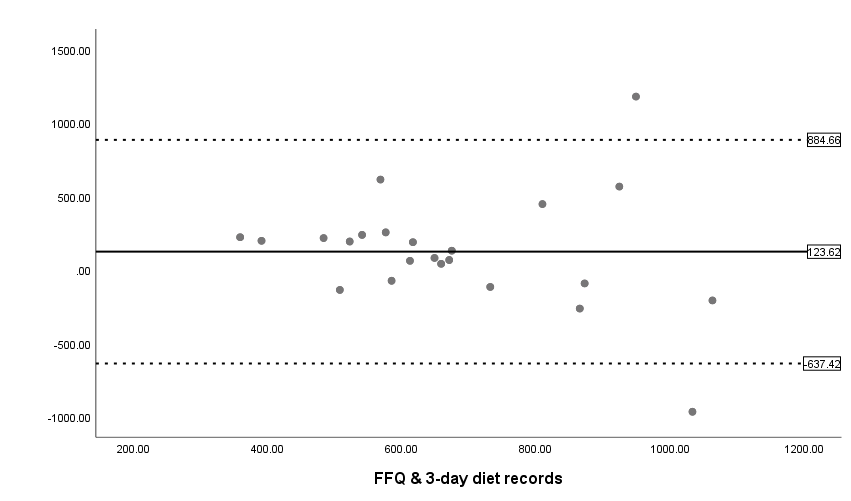 |

| 1. **Iron** |
| --- |
| **Total Sample**  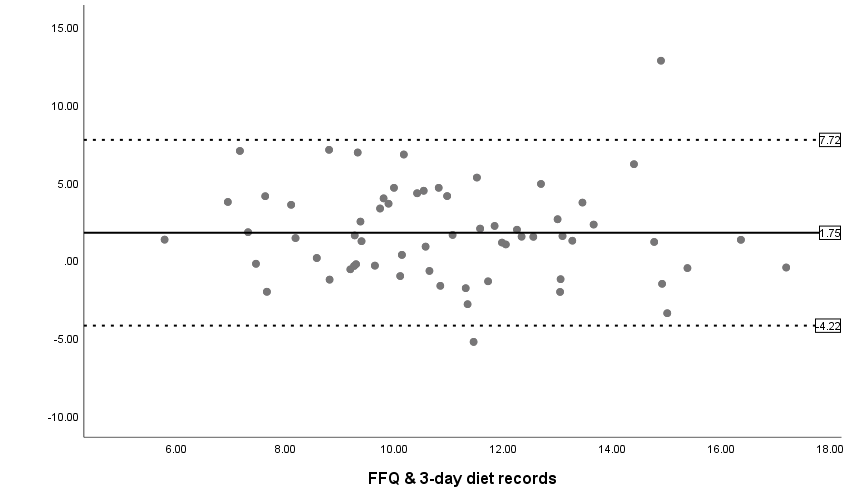 |
| **Female**  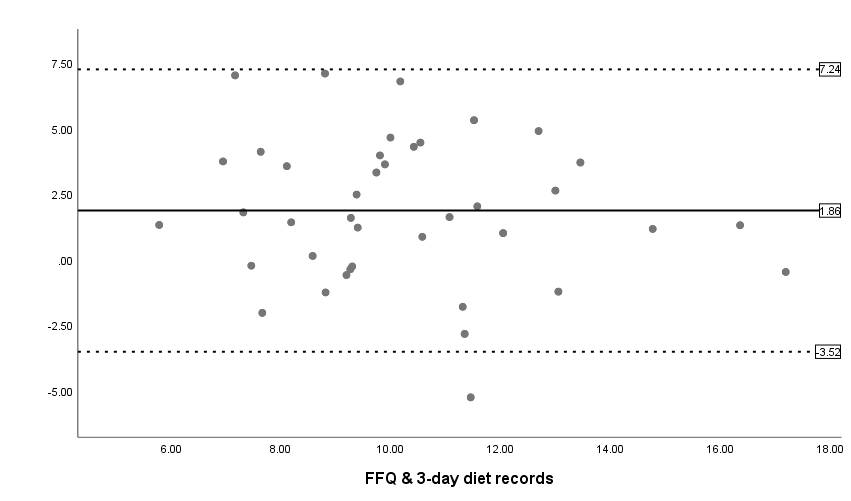 |
| **Male**  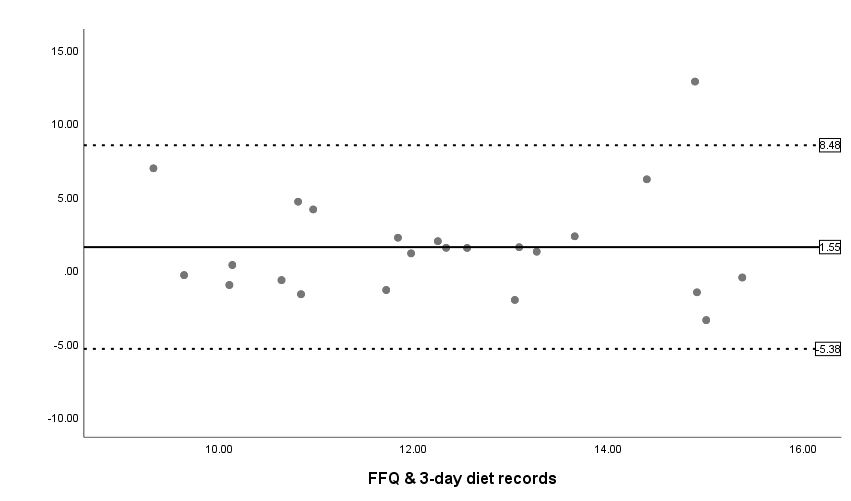 |

| 1. **Total sugar** |
| --- |
| **Total Sample**  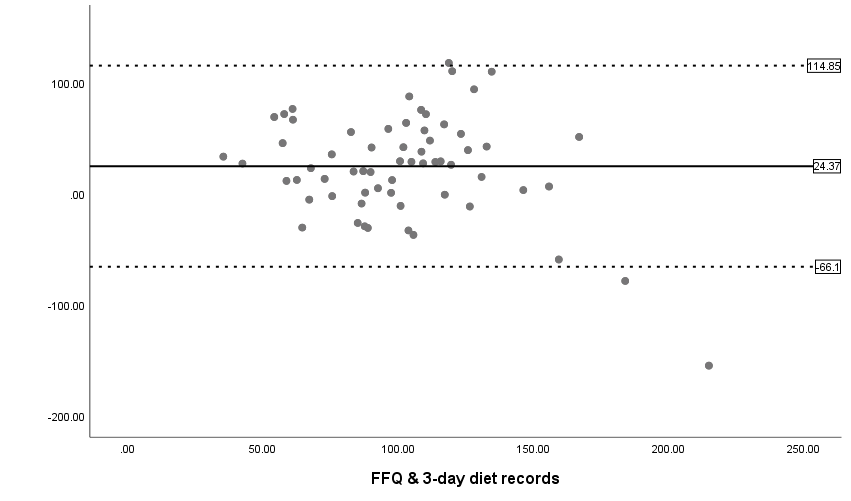 |
| **Female**  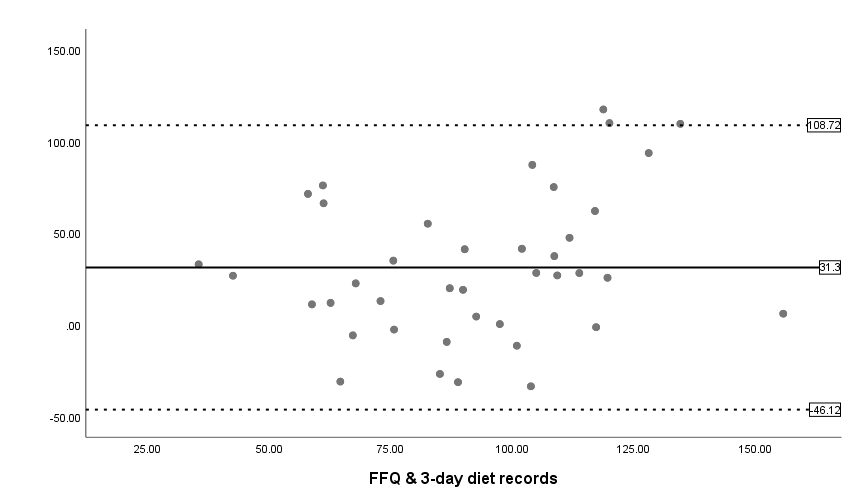 |
| **Male**  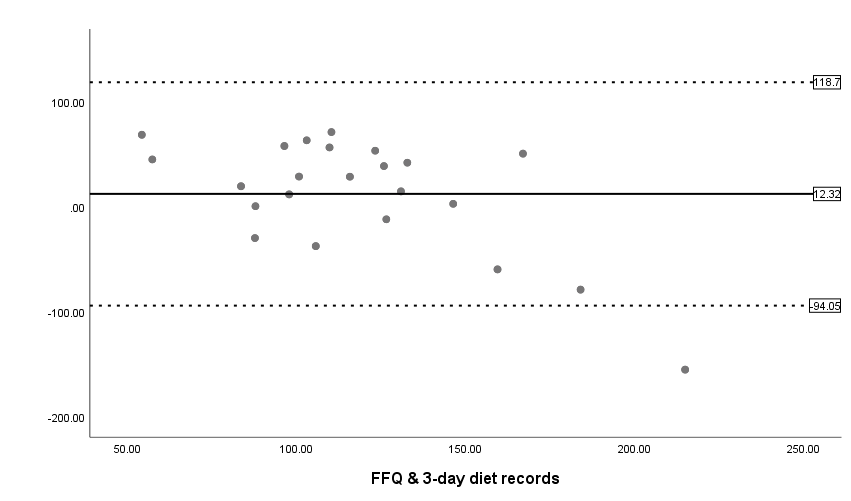 |
